# Development and validation of a pre-trained language model for neonatal morbidities: a retrospective, multicentre, prognostic study

**DOI:** 10.1016/j.landig.2025.100926

**Published:** 2025-12-18

**Authors:** Feng Xie, Philip Chung, Jonathan D Reiss, Erico Tjoa, Davide De Francesco, Thanaphong Phongpreecha, William Haberkorn, Dipro Chakraborty, Alan Lee Chang, Tomin James, Yeasul Kim, Samson Mataraso, Camilo Espinosa, Liu Yang, Chi-Hung Shu, Lei Xue, Eloïse Berson, Neshat Mohammadi, Sayane Shome, S Momsen Reincke, Marc Ghanem, Ivana Maric, Brice Gaudilliere, Martin S Angst, Karl Sylvester, Gary M Shaw, Lawrence S Prince, David K Stevenson, Nima Aghaeepour

**Affiliations:** a Department of Anesthesiology, Perioperative and Pain Medicine, Stanford University, Stanford, CA, USA; b Department of Pediatrics, Stanford University School of Medicine, Stanford, CA, USA; c Department of Biomedical Data Science, Stanford University, Stanford, CA, USA; d Division of Computational Health Sciences, Department of Surgery, University of Minnesota, Minneapolis, MN, USA; e Department of Surgery, Stanford University, Stanford, CA, USA

## Abstract

**Background:**

Early identification and monitoring of neonatal morbidities are critical for timely interventions that can prevent complications, optimise resource use, and support families. Although traditional tools based on tabular data and biomarkers are beneficial, they are restricted in assessing the risk of morbidities in newborns. In this study, we developed NeonatalBERT, a pre-trained large language model (LLM) that estimates the risk of neonatal morbidities from clinical notes.

**Methods:**

This prognostic study investigated retrospective primary and external cohorts from two different quaternary-care academic medical centres in the USA: Stanford Health Care and Beth Israel Deaconess Medical Center. NeonatalBERT was initially pre-trained on clinical notes from the primary cohort and then fine-tuned separately for both cohorts. NeonatalBERT was also compared against other existing LLMs, such as BioBERT and Bio-ClinicalBERT, as well as traditional machine learning and logistic regression models using tabular features. NeonatalBERT was evaluated on 19 neonatal morbidities (respiratory distress syndrome, bronchopulmonary dysplasia, pulmonary haemorrhage, pulmonary hypertension, atelectasis, aspiration syndrome, intraventricular haemorrhage, periventricular leukomalacia, neonatal seizures, other CNS disorders, patent ductus arteriosus, cardiovascular instability, sepsis, candidiasis, anaemia, jaundice, necrotising enterocolitis, retinopathy of prematurity, and death) for the primary cohort and ten for the external cohort (respiratory distress syndrome, bronchopulmonary dysplasia, pulmonary haemorrhage, intraventricular haemorrhage, patent ductus arteriosus, sepsis, jaundice, necrotising enterocolitis, retinopathy of prematurity, and death). For each outcome, the area under the receiver operating characteristic curve, area under the precision-recall curve (AUPRC), and F1 scores were evaluated.

**Findings:**

32 321 newborns were included in the primary cohort, including 27 411 in the primary training set (mean gestational age 38·64 weeks [SD 2·30]; 13 056 [47·6%] female and 14 355 [52·4%] male newborns) and 4910 in the primary testing set (mean gestational age 38·64 [2·13] weeks; 2336 [47·6%] female and 2574 [52·4%] male newborns). Additionally, 7061 newborns were selected into the external cohort, including 5653 in the external training set (1567 [27·7%] premature and 4086 [72·3%] term births; 2614 [46·2%] female and 3039 [53·8%] male newborns) and 1408 in the external testing set (383 [27·2%] premature and 1025 [72·8%] term births; 624 [44·3%] female and 784 [55·7%] male newborns). In the primary cohort, the mean AUPRC over 19 outcomes was 0·291 (95% CI 0·268–0·314) for NeonatalBERT, 0·238 (0·217–0·259) for Bio-ClinicalBERT, 0·217 (0·197–0·236) for BioBERT, and 0·194 (0·177–0·211) for the traditional model using tabular data. In the external cohort, NeonatalBERT had a mean AUPRC of 0·360 (0·328–0·393), outperforming other models with the range of 0·224–0·333.

**Interpretation:**

Based on validation using two large-scale US datasets, NeonatalBERT effectively estimates the risk of neonatal morbidities from unstructured clinical notes of newborns. The promising results from this study show the potential of NeonatalBERT to enhance neonatal care and streamline hospital operations.

**Funding:**

National Institutes of Health, Burroughs Wellcome Fund, March of Dimes Foundation, Alfred E Mann Foundation, Gates Foundation, Christopher Hess Research Fund, Roberts Foundation Research Fund, Prematurity Research Center, and Stanford Maternal & Child Health Research Institute Postdoctoral Support funds.

## Introduction

The neonatal period is a critical juncture in human development, involving substantial physiological adjustments as newborns transition to life outside the womb. Despite medical advances, over 1 million neonatal deaths occur annually worldwide.^1^ Early prediction of neonatal conditions is pivotal for initiating life-saving interventions, with validated tools estimating risks for common prematurity-related outcomes^2^ such as mortality, sepsis, neurodevelopmental impairments, hyperbilirubinaemia, and bronchopulmonary dysplasia.

**Research in context**

**Evidence before this study**

We searched PubMed between Jan 1, 2015, and June 1, 2025, with the search terms ("neonatal" OR "newborn") AND ("predict" OR "prediction" OR "estimate") AND ("machine learning" OR "regression" OR "deep learning" OR "transformer" OR "language model"), with no language restrictions. Existing literature showed that traditional machine learning models—often based on structured data—are constrained in predictive performance due to their inability to incorporate the context-rich information embedded in unstructured clinical notes. Although transformer-based models, including BERT variants, have improved predictions in general clinical domains, we found no previous studies that developed or validated a foundation model specifically pre-trained in a neonatal context. Furthermore, few studies explored large-scale, note-based prediction of neonatal morbidities across diverse hospital settings.

**Added value of this study**

This study introduces NeonatalBERT, a domain-specific language model pre-trained on neonatal clinical notes. Unlike general clinical language models, NeonatalBERT captures nuanced neonatal-specific terminologies and clinical patterns. The probabilistic outputs of NeonatalBERT enable early risk estimation of neonatal morbidities, including those that might not yet be clinically apparent, supporting timely intervention and decision making. Through evaluation across internal and external cohorts, we show the feasibility of using unstructured notes for early risk estimation, such as bronchopulmonary dysplasia, necrotising enterocolitis, sepsis, and mortality.

**Implications of all the available evidence**

NeonatalBERT enables early prediction of neonatal morbidities by leveraging unstructured clinical notes, offering timely insights to support clinical decision making. Although the predictive value of NeonatalBERT varies by outcome—being more immediate for conditions identified at birth and anticipatory for those with delayed onset—its consistent performance across cohorts shows its potential to complement existing tools, especially when structured data are incomplete or unavailable.

Advancements in machine learning and artificial intelligence (AI) methods, combined with the availability of large-scale electronic health records (EHRs), have enhanced our ability to predict life-impairing neonatal conditions, enabling clinicians to initiate appropriate clinical care.^3^ Traditionally, structured EHR data such as laboratory studies, tests, and vital signs have been used to inform neonatal complications,^2^ yet unstructured clinical notes remain underexploited in neonatal care. These notes capture nuanced, domain-specific details, such as physician observations and inter-provider communications, which are not typically found in structured data.

Unstructured clinical notes data are a great source of patient information but leveraging them for disease prediction is challenging due to the irregularity of free text and the complexity of modelling natural human language. Advances in natural language processing, particularly contextual word embedding models and large language models (LLMs)^4^ have shown remarkable capabilities in natural language understanding in health care.^5^ Pre-trained language models, such as Bidirectional Encoder Representations from Transformers (BERT),^6^ have shown strong language understanding that can be used for a multitude of text prediction tasks. BERT has been extended to BioBERT^7^ and Bio-ClinicalBERT^8^ for biomedical text and clinical narratives, respectively. Although Bio-ClinicalBERT has been used for disease prediction in adults,^9^ neonatal diseases and conditions affecting newborns are different from adult diseases, thus both disease terminology and documentation of clinical examinations and testing have different linguistic characteristics from clinical text for adult patients.

In this study, we introduce NeonatalBERT, a BERT-based model specifically designed to use unstructured clinical notes to predict various neonatal morbidities. By adapting language models for the neonatal context, NeonatalBERT shows how a pre-trained language model can enhance the early detection of neonatal morbidities.

## Methods

### Study design and cohort

This is a prognostic study using retrospective EHRs associated with routine clinical care of newborns from two large academic medical centres in the USA. The study was approved by the Stanford University Institutional Review Board (approval number 39225); the board determined that informed consent was not required for the secondary use of EHR data. The study adhered to Transparent Reporting of a Multivariable Prediction Model for Individual Prognosis or Diagnosis (TRIPOD) reporting guidelines.^10^ Patients and the public were not involved in the design, conduct, reporting, or dissemination of this study.

### Data extraction and outcomes

**Figure 1:**
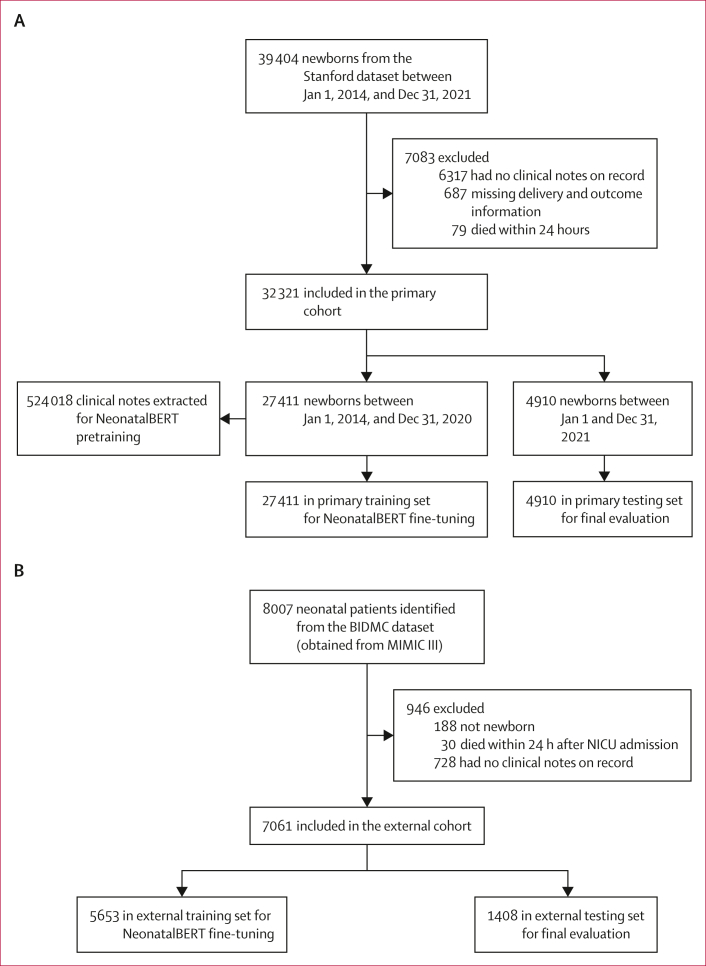
Formation of the primary cohort and external cohort (A) The primary cohort from Stanford-affiliated hospitals. (B) The external cohort from the BIDMC. BERT=Bidirectional Encoder Representations from Transformers. BIDMC=Beth Israel Deaconess Medical Center. MIMIC III=Medical Information Mart for Intensive Care III. NICU=neonatal intensive care unit.

The primary cohort consisted of all babies born between Jan 1, 2014, and Dec 31, 2021, at Stanford-affiliated hospitals and clinics from the Stanford Research Data Repository (figure 1A). Babies born between Jan 1, 2014, and Dec 31, 2020, were assigned to the primary training set, and newborns born between Jan 1 and Dec 31, 2021, were assigned to the primary testing set. This temporal validation design was chosen to be consistent with future application scenarios and to evaluate whether population shift would affect the performance of the model. The 19 neonatal outcomes of interest cover respiratory morbidities (respiratory distress syndrome, bronchopulmonary dysplasia, pulmonary haemorrhage, pulmonary hypertension, atelectasis, aspiration syndrome), neurological morbidities (intraventricular haemorrhage, periventricular leukomalacia, neonatal seizures, and other CNS disorders), cardiac morbidities (patent ductus arteriosus and cardiovascular instability), infectious diseases (sepsis and candidiasis), blood disorders (anaemia and jaundice), and other conditions (necrotising enterocolitis, retinopathy of prematurity, and death). We extracted all clinical notes from these patients, including history and physical notes, progress notes, procedure notes, general notes, and consultation summaries. Maternal and prenatal history were derived from documentation within neonatal clinical notes.

The external cohort consisted of newborn records from Beth Israel Deaconess Medical Center extracted from the Medical Information Mart for Intensive Care III database^11^ compiled by the Massachusetts Institute of Technology Laboratory for Computational Physiology (figure 1B). The external cohort included ten primary outcomes: respiratory distress syndrome, bronchopulmonary dysplasia, pulmonary haemorrhage, intraventricular haemorrhage, patent ductus arteriosus, sepsis, jaundice, necrotising enterocolitis, retinopathy of prematurity, and death. Because reliable dates were unavailable due to date shifting from the de-identification process, the entire external cohort was randomly divided at the patient level into two non-overlapping datasets: an external training set (80%) and an external testing set (20%). For the primary cohort, Systematized Medical Nomenclature for Medicine–Clinical Terminology codes were used to identify the presence or absence of each outcome (appendix pp 2–4). For the external cohort, ICD-9 codes^12^ were used for outcome identification (appendix p 5). Patients without clinical notes, missing outcome data, or who died within 24 h of birth were excluded.

### Model development and statistical analysis

**Figure 2:**
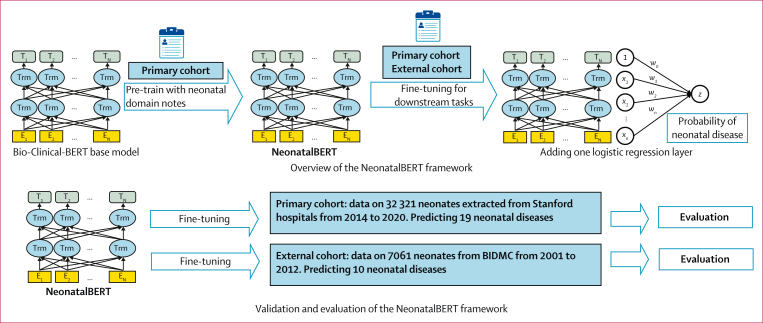
Development and validation of NeonatalBERT BERT=Bidirectional Encoder Representations from Transformers. BIDMC=Beth Israel Deaconess Medical Center.

We developed NeonatalBERT by domain-adaptive pre-training based on Bio-ClinicalBERT,^8^ with a corpus of neonatal clinical notes obtained during the first 3 months of birth from the training set of the primary cohort to contextualise the model to the language of neonatal medicine. The domain-adaptive pre-training used the same self-supervised objectives as the original BERT model, which includes masked language modelling and next-sentence prediction. To determine the best-performing model, we used a manual method and used a subset of the training data for hyperparameter tuning, effectively serving as a validation set within the primary cohort. NeonatalBERT was further fine-tuned to perform neonatal outcome prediction for each cohort by adding a logistic classification layer for each outcome in the respective cohort’s dataset. Fine-tuning is the process of adapting a pre-trained model to a specific task, such as estimating the risk of a specific neonatal outcome. Only clinical notes within the first day after birth from the respective cohort’s training set were used for fine-tuning to ensure the risk estimate was of clinical utility. This yielded two separate fine-tuned NeonatalBERT models—one for the primary cohort and one for the external cohort. The fine-tuned models were evaluated using the testing set corresponding to each cohort (figure 2). The predictive power of NeonatalBERT was measured using the area under the receiver operating characteristic curve (AUROC) and the area under the precision-recall curve (AUPRC). While AUROC is based on recall and the true positive rate, AUPRC is based on recall and positive predictive value. Hence, AUPRC is more representative when outcomes are rare and unbalanced, like most neonatal outcomes. For visualisation, we reported the relative change in AUPRC compared with a no-skill classifier, since AUPRC is prevalence-dependent. Considering the imbalance of most neonatal outcomes (table 1), the F1 score^13^ was calculated to provide another meaningful assessment.

**Table 1:** Characteristics of the primary and external cohorts

	Primary cohort (Stanford)			External cohort (BIDMC)		
	Overall (n=32 321)	Training set (2014–20 data; n=27 411)	Testing set (2021 data; n=4910)	Overall (n=7061)	Training set (randomly split 80%; n=5653)	Testing set (randomly split 20%; n=1408)
Mother’s age, years	32·62 (5·59)	32·53 (5·62)	33·13 (5·37)	··	··	··
Gestational age, weeks	38·64 (2·28)	38·64 (2·30)	38·64 (2·13)	··	··	··
Premature birth (<37 weeks)	4775 (14·8)	4075 (14·9%)	700 (14·3%)	1950 (27·6%)	1567 (27·7%)	383 (27·2%)
Extremely preterm (<28 weeks)	228 (0·7%)	254 (0·9%)	34 (0·7%)	··	··	··
Very preterm (28 weeks to <32 weeks)	466 (1·4%)	411 (1·5%)	55 (1·1%)	··	··	··
Moderate to late preterm (32 weeks to <37 weeks)	4021 (12·4%)	3410 (12·4%)	611 (12·4%)	··	··	··
Term birth (≥37 weeks)	27 546 (85·2%)	23 336 (85·1%)	4210 (85·7%)	5111 (72·4%)	4086 (72·3%)	1025 (72·8%)
Birthweight, g	3127·91 (564·05)	3121·59 (544·63)	3163·23 (661·04)	··	··	··
Sex						
Male	16 929 (52·4%)	14 355 (52·4%)	2574 (52·4%)	3823 (54·1%)	3039 (53·8%)	784 (55·7%)
Female	15 392 (47·6%)	13 056 (47·6%)	2336 (47·6%)	3238 (45·9%)	2614 (46·2%)	624 (44·3%)
Ethnicity						
White	10 399 (32·2%)	8954 (32·7%)	1445 (29·4%)	4299 (60·9%)	3466 (61·3%)	833 (59·2%)
Hispanic	9724 (30·1%)	8337 (30·4%)	1387 (28·2%)	352 (5·0%)	285 (5·0%)	67 (4·8%)
Asian	8763 (27·1%)	7195 (26·2%)	1568 (31·9%)	669 (9·5%)	522 (9·2%)	147 (10·4%)
African	693 (2·1%)	596 (2·2%)	97 (2·0%)	822 (11·6%)	649 (11·5%)	173 (12·3%)
Other	2742 (8·5%)	2329 (8·5%)	413 (8·4%)	919 (13·0%)	731 (12·9%)	188 (13·4%)
Respiratory morbidities						
Respiratory distress syndrome	2564 (7·9%)	2249 (8·2%)	315 (6·4%)	1264 (17·9%)	1031 (18·2%)	233 (16·5%)
Bronchopulmonary dysplasia	318 (1·0%)	282 (1·0%)	36 (0·7%)	160 (2·3%)	133 (2·4%)	27 (1·9%)
Pulmonary haemorrhage	50 (0·2%)	46 (0·2%)	4 (0·1%)	20 (0·3%)	16 (0·3%)	4 (0·3%)
Pulmonary hypertension	182 (0·6%)	152 (0·6%)	30 (0·6%)	··	··	··
Atelectasis	328 (1·0%)	305 (1·1%)	23 (0·5%)	··	··	··
Aspiration syndrome	401 (1·2%)	356 (1·3%)	45 (0·9%)	··	··	··
Neurological morbidities						
Intraventricular haemorrhage	292 (0·9%)	256 (0·9%)	36 (0·7%)	184 (2·6%)	151 (2·7%)	33 (2·3%)
Periventricular leukomalacia	28 (0·1%)	24 (0·1%)	4 (0·1%)	··	··	··
Seizures	135 (0·4%)	116 (0·4%)	19 (0·4%)	··	··	··
Other CNS disorders	1016 (3·1%)	844 (3·1%)	172 (3·5%)	··	··	··
Cardiac morbidities						
Patent ductus arteriosus	1087 (3·4%)	948 (3·5%)	139 (2·8%)	370 (5·2%)	306 (5·4%)	64 (4·5%)
Cardiovascular instability	2021 (6·3%)	1775 (6·5%)	246 (5·0%)	··	··	··
Infectious diseases						
Neonatal sepsis	602 (1·9%)	560 (2·0%)	42 (0·9%)	199 (2·8%)	159 (2·8%)	40 (2·8%)
Candidiasis	220 (0·7%)	186 (0·7%)	34 (0·7%)	··	··	··
Blood disorders						
Anaemia	858 (2·7%)	769 (2·8%)	89 (1·8%)	··	··	··
Jaundice	15 192 (47·0%)	13 083 (47·7%)	2109 (43·0%)	2600 (36·8%)	2066 (36·5%)	534 (37·9%)
Other conditions						
Necrotising enterocolitis	92 (0·3%)	81 (0·3%)	11 (0·2%)	58 (0·8%)	46 (0·8%)	12 (0·9%)
Retinopathy of prematurity	638 (2·0%)	576 (2·1%)	62 (1·3%)	147 (2·1%)	118 (2·1%)	29 (2·1%)
Neonatal death	148 (0·5%)	125 (0·5%)	23 (0·5%)	35 (0·5%)	31 (0·5%)	4 (0·3%)

Data are mean (SD) or n (%). BIDMC=Beth Israel Deaconess Medical Center.

Baseline comparison models were created for each cohort using logistic regression on only tabular EHR features from the first day of birth. The list of tabular variables is given in the appendix (appendix p 2). We also compared the NeonatalBERT with the previously developed BERT-based language models, including BioBERT and Bio-ClinicalBERT. The baseline models were trained and evaluated on the same patients and their notes in each cohort as those used to fine-tune NeonatalBERT. We also included a comparison with conversation-based LLMs (eg, Meta Llama-3.1-8B) to show the improved utility of our model in clinical risk estimation and decision making support. Implementation details regarding data processing, model training, and parameter tuning are provided in the appendix (appendix pp 16–19).

### Role of the funding source

The funders of the study had no role in study design, data collection, data analysis, data interpretation, or writing of the report.

## Results

32 321 newborns were included in the primary cohort, including 27 411 in the primary training set (mean gestational age 38·64 weeks [SD 2·30]; 13 056 [47·6%] female and 14 355 [52·4%] male newborns) and 4910 in the primary testing set (mean gestational age 38·64 [2·13] weeks; 2336 [47·6%] female and 2574 [52·4%] male newborns; table 1). The study population included both term and preterm neonates, allowing the model to learn patterns across a broad clinical spectrum. Neonatal morbidities varied in both prevalence and gestational age dependency. Additionally, 7061 newborns were selected into the external cohort, including 5653 in the external training set (1567 [27·7%] premature and 4086 [72·3%] term births; 2614 [46·2%] female and 3039 [53·8%] male newborns) and 1408 in the external testing set (383 [27·2%] premature and 1025 [72·8%] term births; 624 [44·3%] female and 784 [55·7%] male newborns). Compared with those in the primary cohort, newborns in the external cohort had a higher frequency of neonatal morbidities as they were neonatal intensive care admissions with higher premature rates.

NeonatalBERT showed high performance in estimating the risk of 19 neonatal conditions in the primary cohort (table 2; appendix pp 6–9). The average AUROC over 19 outcomes was 0·910 (95% CI 0·900–0·921) for NeonatalBERT, 0·884 (0·870–0·901) for Bio-ClinicalBERT, 0·877 (0·867–0·890) for BioBERT, and 0·775 (0·749–0·802) for the traditional regression model using tabular data. NeonatalBERT also had an AUPRC of 0·291 (95% CI 0·268–0·314), outperforming Bio-ClinicalBERT (0·238, 95% CI 0·217–0·259), BioBERT (0·217, 95% CI 0·197–0·236), and the traditional regression model using tabular data (0·194, 0·177–0·211). The AUPRC was up to 129·6 times higher than that of a random classifier for mortality prediction, 13·5 times higher for periventricular leukomalacia, 14·1 times higher for patent ductus arteriosus, 25·7 times higher for intraventricular haemorrhage, and 88·2 times higher for pulmonary haemorrhage. Compared with AUROC, the difference in AUPRC was more pronounced, indicating that NeonatalBERT outperformed baselines in classifying unbalanced rare events. The Llama-based conversational LLMs did even worse in terms of sensitivity and specificity (appendix pp 10).

**Table 2:** Comparison of performance metrics achieved by different models on the testing data from the primary cohort and the external cohort

	AUPRC (absolute)				AUPRC (× baseline)∗			
	NeonatalBERT	Logistic regression†	Bio-ClinicalBERT	Bio-BERT	NeonatalBERT	Logistic regression†	Bio-ClinicalBERT	Bio-BERT
**Primary cohort (Stanford)**								
Respiratory morbidities								
Respiratory distress syndrome	0·707 (0·650–0·763)	0·436 (0·384–0·493)	0·587 (0·532–0·643)	0·371 (0·322–0·423)	11·0	6·8	9·2	5·8
Bronchopulmonary dysplasia	0·476 (0·338–0·618)	0·468 (0·318–0·614)	0·388 (0·283–0·492)	0·296 (0·238–0·442)	47·6	46·8	38·8	29·6
Pulmonary haemorrhage	0·088 (0·029–0·344)	0·078 (0·005–0·283)	0·026 (0·011–0·058)	0·025 (0·009–0·072)	88·2	78·0	26·0	25·0
Pulmonary hypertension	0·151 (0·086–0·254)	0·015 (0·005–0·072)	0·108 (0·067–0·1900)	0·108 (0·067–0·190)	25·2	2·5	18·0	18·0
Atelectasis	0·06 (0·028–0·141)	0·017 (0·008–0·042)	0·062 (0·021–0·200)	0·089 (0·022–0·247)	12·0	3·4	12·4	17·8
Aspiration syndrome	0·097 (0·061–0·183)	0·021 (0·015–0·034)	0·057 (0·032–0·089)	0·063 (0·025–0·101)	10·8	2·3	6·3	7·0
Neurological morbidities								
Intraventricular haemorrhage	0·180 (0·129–0·286)	0·129 (0·086–0·199)	0·127 (0·089–0·195)	0·131 (0·085–0·178)	25·8	18·4	18·1	18·7
Periventricular leukomalacia	0·014 (0·003–0·036)	0·004 (0·000–0·018)	0·009 (0·001–0·029)	0·010 (0·002–0·030)	13·5	4·0	9·0	10·0
Seizures	0·083 (0·042–0·177)	0·033 (0·010–0·085)	0·161 (0·066–0·318)	0·139 (0·052–0·287)	21·5	8·3	40·3	34·8
Other CNS disorders	0·208 (0·051–0·488)	0·003 (0·001–0·007)	0·188 (0·051–0·361)	0·242 (0·047–0·461)	6·0	0·1	5·4	6·9
Cardiac morbidities								
Patent ductus arteriosus	0·395 (0·318–0·500)	0·144 (0·090–0·210)	0·301 (0·239–0·364)	0·400 (0·326–0·466)	14·1	5·1	10·8	14·3
Cardiovascular instability	0·399 (0·339–0·466)	0·274 (0·239–0·322)	0·256 (0·214–0·286)	0·234 (0·194–0·263)	8·0	5·5	5·1	4·7
Infectious diseases								
Neonatal sepsis	0·159 (0·093–0·291)	0·072 (0·033–0·140)	0·242 (0·156–0·358)	0·129 (0·108–0·205)	17·7	8·0	26·9	14·3
Candidiasis	0·014 (0·009–0·023)	0·012 (0·007–0·035)	0·011 (0·008–0·015)	0·009 (0·007–0·014)	2·0	1·7	1·6	1·3
Blood disorders								
Anaemia	0·627 (0·549–0·727)	0·552 (0·442–0·655)	0·551 (0·461–0·633)	0·566 (0·510–0·677)	34·8	30·7	30·6	31·4
Jaundice	0·576 (0·560–0·602)	0·531 (0·507–0·555)	0·55 (0·538–0·574)	0·545 (0·529–0·560)	1·3	1·2	1·3	1·3
Other conditions								
Necrotising enterocolitis	0·093 (0·027–0·204)	0·064 (0·030–0·155)	0·175 (0·061–0·435)	0·093 (0·055–0·239)	46·4	32·0	87·5	46·5
Retinopathy of prematurity	0·555 (0·437–0·679)	0·447 (0·373–0·553)	0·349 (0·233, 0·443)	0·35 (0·218–0·443)	27.7	22.4	17.5	17.5
Neonatal death	0·648 (0·498–0·780)	0·392 (0·249–0·548)	0·372 (0·226–0·526)	0·315 (0·198–0·441)	129.6	78.4	74.4	63.0
Average over all outcomes	0·291 (0·268–0·314)	0·194 (0·177–0·211)	0·238 (0·217–0·259)	0·217 (0·197–0·236)	28·6	18·7	23·1	19·4
**External cohort (Beth Israel Deaconess Medical Center)**								
Respiratory morbidities								
Respiratory distress syndrome	0·806 (0·760–0·855)	0·552 (0·487–0·633)	0·802 (0·757–0·849)	0·793 (0·748–0·846)	4·5	3·1	4·5	4·4
Bronchopulmonary dysplasia	0·263 (0·207–0·362)	0·12 (0·073–0·22)	0·244 (0·179–0·374)	0·238 (0·182–0·357)	11·5	5·2	10·6	10·4
Pulmonary haemorrhage	0·07 (0·035–0·155)	0·019 (0·001–0·102)	0·037 (0·016–0·085)	0·047 (0·016–0·156)	23·3	6·3	12·4	15·5
Neurological morbidities								
Intraventricular haemorrhage	0·327 (0·241–0·472)	0·181 (0·090–0·310)	0·251 (0·196–0·351)	0·272 (0·189–0·382)	12·6	7·0	9·7	10·5
Cardiac morbidities								
Patent ductus arteriosus	0·44 (0·359–0·525)	0·262 (0·200–0·348)	0·419 (0·346–0·508)	0·417 (0·33–0·507)	8·5	5·0	8·1	8·0
Infectious diseases								
Neonatal sepsis	0·201 (0·125–0·280)	0·225 (0·147–0·367)	0·188 (0·121–0·264)	0·162 (0·102–0·238)	7·2	8·0	6·7	5·8
Blood disorders								
Jaundice	0·837 (0·810–0·856)	0·715 (0·679–0·756)	0·814 (0·780–0·843)	0·809 (0·775–0·837)	2·3	1·9	2·2	2·2
Other conditions								
Necrotising enterocolitis	0·049 (0·034–0·109)	0·038 (0·019–0·093)	0·071 (0·023–0·217)	0·108 (0·021–0·238)	6·2	4·7	8·8	13·5
Retinopathy of prematurity	0·344 (0·251–0·489)	0·112 (0·077–0·202)	0·301 (0·230–0·444)	0·289 (0·214–0·412)	16·4	5·3	14·3	13·8
Death	0·267 (0·117–0·563)	0·021 (0·006–0·079)	0·146 (0·069–0·309)	0·193 (0·084–0·435)	53·5	4·1	29·2	38·6
Mean over all outcomes	0·360 (0·328–0·393)	0·224 (0·202–0·247)	0·327 (0·301–0·353)	0·333 (0·303–0·363)	14·6	5·1	10·6	12·3

Data are presented as estimates with 95% CIs. AUPRC=area under the precision-recall curve.

∗ Represents relative increase or decrease in AUPRC compared with a random classifier (baseline).

† Logistic regression using tabular variables only.

Externally, NeonatalBERT maintained high performance (table 2; appendix pp 6–9), with an average AUROC of 0·935 (95% CI 0·928–0·942) and an average AUPRC of 0·360 (0·328–0·393) over all available outcomes. The logistic regression model using only tabular data had an average AUROC of 0·816 (0·772–0·860) and an average AUPRC of 0·224 (0·202–0·247). In comparison, BioBERT and Bio-ClinicalBERT had AUROCs of 0·925 (0·917–0·933) and 0·925 (0·917–0·932), and AUPRCs of 0·333 (0·303–0·363) and 0·327 (0·301–0·353), respectively. In addition, NeonatalBERT had a mean F1 score of 0·416, outperforming logistic regression (0·267), BioBERT (0·379), and Bio-ClinicalBERT (0·386). These findings indicate the potential for the NeonatalBERT to generalise across different hospitals, patient populations, and EHR systems.

**Figure 3:**
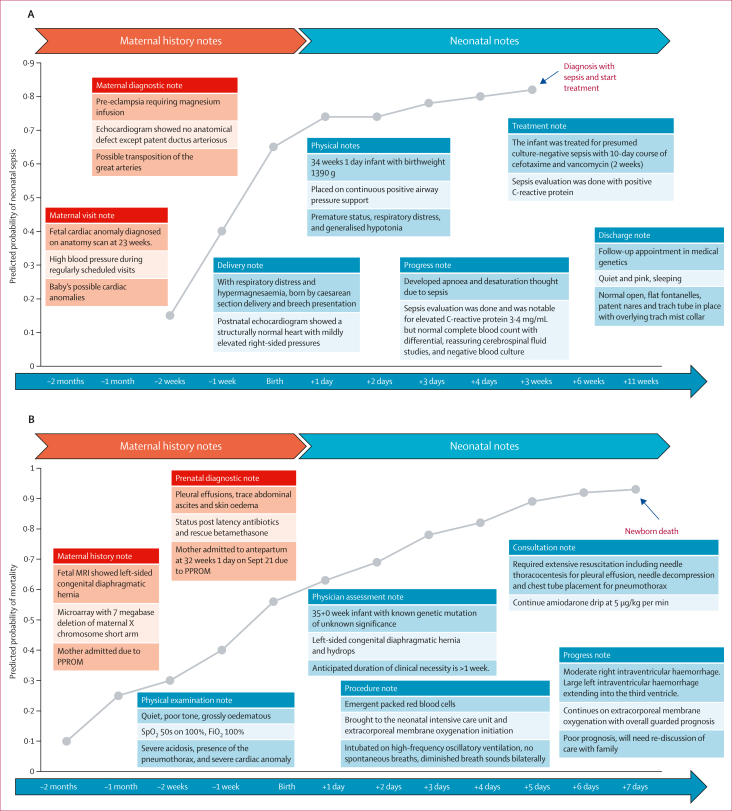

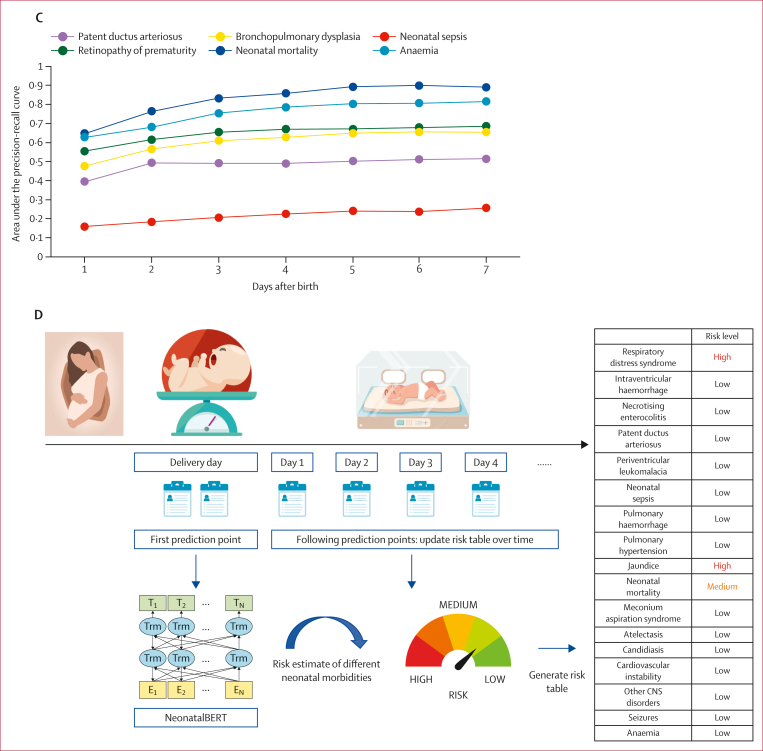
NeonatalBERT supporting clinical decision making in neonatal care Example prediction timeline for neonatal sepsis (A) and neonatal death (B), showing predicted probabilities generated by NeonatalBERT at different timepoints based on clinical notes from the newborn’s record. As new clinical notes are added during the birth and postnatal period, the model iteratively incorporates this information, resulting in improved predictive performance over time. (C) Improvement in prediction performance over time for neonatal outcomes as more longitudinal data become available. (D) Risk stratification dashboard designed to support clinical decision making by providing clinicians with visualised risk estimates and trends. BERT=Bidirectional Encoder Representations from Transformers. PPROM=preterm prelabour rupture of membranes.

*t*-distributed stochastic neighbour embedding analysis revealed distinct clustering of neonatal conditions in two-dimensional plots for both the primary and external cohorts, providing insight into the rationale behind prediction accuracy (appendix pp 20–21). Newborns with various neonatal morbidities, including respiratory distress syndrome, patent ductus arteriosus, intraventricular haemorrhage, necrotising enterocolitis, and sepsis showed cohesive clustering, whereas newborns with jaundice did not form a clearly defined cluster, thereby offering a view into the discrepancies in prediction accuracy. Figure 3 shows potential clinical use cases for supporting clinical decision making in practice for newborns at birth. Prediction is shown for an individual patient born at 35 weeks gestational age, incorporating keywords in this newborn’s notes across pre-birth and post-birth days to formulate predictions on sepsis. Importantly, the model synthesised a broad range of information, including maternal history, subtle clinical signs, and laboratory data, enabling flagging of elevated sepsis risk well before formal diagnosis in the third week of life. The figure also shows prediction for another individual newborn for neonatal mortality. Although such recorded diagnoses are often perceived as being highly accurate, recent studies^14^ report survival rates of 60–80% after a prediction of death, reflecting wide prognostic variability. The ability of NeonatalBERT to produce individualised, early risk estimates in such complex cases highlights the potential to support timely clinical decisions and family counselling. Performance using clinical notes from day 1 to day 7 is shown in figure 3 and the appendix (pp 11–12). This analysis supported increased predictive performance when more clinical notes were available and a greater number of clinical observations had accumulated since birth, with average AUPRC improving from 0·291 on day 1, to 0·380 on day 3, to 0·417 on day 7. The performance of NeonatalBERT using different note types was also calculated based on the primary cohort as shown in the appendix (p 22), showing that use of different notes together could improve performance compared with using specific note types only. The model calibration plots are provided in the appendix (p 23).

## Discussion

In this study, we developed NeonatalBERT and showed its performance in across hospitals and populations. Unlike traditional models using only tabular data to predict neonatal morbidities, NeonatalBERT leverages transformer neural networks to distil insights from the rich unstructured medical narratives in clinical notes, which improves contextual understanding and cross-institution applicability.^15^ NeonatalBERT paves the way for a variety of future applications aimed at enhancing neonatal care and streamlining hospital operations.

There are several reasons why NeonatalBERT outperforms baseline models. The pre-trained nature of NeonatalBERT allows it to capture deep contextual understanding from vast amounts of text data, as a language model gains most of its knowledge during pre-training rather than fine-tuning.^16^ Also, clinical notes are more readily available in routine clinical practice compared with, for example, structured biomarker data, which many patients might be missing. These unstructured notes provide a rich source of information, and enabled NeonatalBERT to leverage a larger pool of clinical observations during pre-training and fine-tuning for neonatal populations compared with structured data. This extensive use of textual data is a key factor in the higher performance of NeonatalBERT over traditional machine learning and regression models using only tabular data, as much of the critical information about maternal and newborn health including physical examinations, radiological reports, physician communication history, and specialty genetic test reports resides in the unstructured portion of the EHR.^17^ NeonatalBERT might also capture information about rare diseases that might not be readily accessible through tabular EHR data. Compared with Bio-ClinicalBERT, NeonatalBERT has been trained to have greater domain specificity to neonatal morbidities and descriptions. The initialisation with Bio-ClinicalBERT helps NeonatalBERT start from a higher point of increased domain relevance compared with general-purpose BERT models and saves a substantial amount of time and effort by avoiding the need to train from scratch. Additionally, NeonatalBERT inherits the robust language modelling capacities of BERT, yet remains finely tuned to neonatal-specific knowledge—a specialisation not inherent in baselines like BioBERT or Bio-Clinical BERT, making it superior in neonatal outcome predictions. While Llama-like LLMs excel in conversational and generative tasks,^18^ their performance falls short in predicting neonatal conditions, as shown in our experiments, consistent with several previous studies.^19^^,^^20^ Moreover, these LLMs face substantial challenges in clinical utility due to their substantial computational resource requirements, even for inference tasks, making them unsuitable for resource-limited health-care settings.^21^ By contrast, BERT-based models strike an optimal balance between predictive performance, computational efficiency, and clinical applicability,^19^^,^^22^ positioning them as a more practical and effective solution for neonatal condition risk estimation.

NeonatalBERT could help identify early risk factors for neonatal morbidities by leveraging clinical notes available before formal diagnosis, supporting timely risk stratification. NeonatalBERT could empower neonatologists with predictive insights and enable the early detection of potential health issues, allowing for prompt interventions that could substantially alter the child's health trajectory. For instance, NeonatalBERT can forecast sepsis in asymptomatic term neonates, who might not show symptoms at delivery, thus facilitating early sepsis evaluations and antibiotic treatment.^23^ NeonatalBERT proved highly effective in predicting sepsis occurrence several days after birth, but the actual diagnosis and treatment took place in the third week. Thus, NeonatalBERT can integrate information and potentially guide an earlier prediction of sepsis than that made by clinicians. From another case of newborn mortality, a high probability of death was predicted during the first few days, followed by actual death at day 7. Hence, NeonatalBERT could be used to help physicians estimate the likelihood of potential adverse events in advance and prepare families for a potentially poor outcome earlier.

Furthermore, the predictive power of NeonatalBERT could aid in early identification of infants at risk for necrotising enterocolitis, which is hard for clinicians to predict.^24^ Early prediction can facilitate timely preventive strategies, such as adjustment of feeding protocols and use of prophylactic probiotics.^25^ For newborns with pulmonary hypertension, the predictions can also prompt timely oxygen support to maintain blood oxygenation and prevent hypoxia. Additionally, by predicting the risk of intraventricular haemorrhage, NeonatalBERT allows for early implementation of strategies to stabilise cerebral blood flow in preterm infants. Such pre-emptive care could minimise the chances of haemorrhage, thereby preventing potential long-term neurological impairments associated with intraventricular haemorrhage.^26^ In cases of respiratory distress syndrome, NeonatalBERT can identify newborns at high risk, enabling the health-care team to prepare for surfactant therapy and respiratory support. Similarly, the ability of NeonatalBERT to predict periventricular leukomalacia on the day of birth allows for recommendation of early diagnostic tests, such as ultrasounds or MRIs for those at high risk, before symptoms manifest. Current decision-support systems, like BiliTool for hyperbilirubinaemia risk assessment, are helpful but restricted and often constrained by data availability.^27^ By contrast, comprehensive analysis of unstructured clinical notes allows NeonatalBERT to predict an array of neonatal conditions with a great degree of flexibility, as the model relies on only unstructured clinical notes to inform diverse disease predictions. Importantly, the utility of NeonatalBERT extends to outcomes that manifest later in the neonatal period, such as bronchopulmonary dysplasia and retinopathy of prematurity. For bronchopulmonary dysplasia, which typically develops after 28 days, early identification of at-risk infants enables tailored interventions, such as optimised ventilatory strategies or the administration of corticosteroids. Similarly, for retinopathy of prematurity, which usually occurs 4–6 weeks postnatally, the predictions of NeonatalBERT could allow for closer ophthalmological monitoring and timely treatment to prevent vision loss.

NeonatalBERT offers objective measures, and a summary risk estimate by analysing all available clinical notes. Neonatal morbidities vary widely in both timing and clinical recognition. Conditions such as meconium aspiration syndrome and respiratory distress syndrome are typically identified at or shortly after birth, whereas other conditions, such as sepsis or necrotising enterocolitis, might emerge days to weeks later. Accordingly, risk estimates might be descriptive for early-onset conditions and predictive for those that develop over time. Although neonatologists are generally able to form an early impression and assess risk likelihood, their evaluations often depend on individual experience and expertise, which can vary.^28^ In this context, the risk estimates of the model offer an additional layer of verification, which is particularly valuable in time-sensitive neonatal settings. Several studies^29^ have highlighted the role of data-driven decision-making support tools to help neonatologists rethink, reassess, and double-check such patients, minimising the risk of missed morbidities or overlooked risk factors. NeonatalBERT can be integrated into existing medical informatics workflows and is a flexible solution for reconceptualising and prototyping clinical notes in neonatal care without tedious data processing and harmonisation.^22^

We propose a workflow where the model generates daily risk estimates for each neonate at the end of clinical shifts. These risk estimates can serve as valuable datapoints for clinicians during morning rounds, helping to inform and refine care plans for the current day. By presenting these risk assessments through a dashboard integrated into the EHR system, clinicians can have streamlined access to actionable insights that complement other clinical data and support more proactive, data-driven decision making during routine patient care.^30^ Implementation of NeonatalBERT in neonatal care necessitates careful consideration of ethical implications, data privacy, and potential biases inherent in clinical documentation.^31^ Clinical notes might reflect variability in provider documentation styles, institutional practices, and demographic representation (including gestational age, sex, and race or ethnicity),^32^ which could inadvertently influence the model predictions. To address these concerns, the model should be further trained on a diverse dataset to mitigate biases and enhance generalisability. Audits and fairness evaluations will be crucial to ensure equity in model predictions across subpopulations. To reduce the risk of feedback loops, NeonatalBERT should be regularly retrained on updated EHR data to reflect evolving documentation. Future research will focus on conducting prospective studies to validate NeonatalBERT in live clinical settings, evaluating effectiveness in improving clinical decision making and patient outcomes. The scalability of NeonatalBERT is also crucial for its practical adoption across diverse health-care settings. In resource-rich environments, further pre-training on local datasets can account for these site-specific differences and enhance domain-specific adaptation.^33^ For resource-limited settings, NeonatalBERT can operate in fine-tuning-only mode, analysing clinical notes without additional computational demands.

This study has several limitations. First, the dataset was based on an electronic database with routinely collected data for clinical care. Thus, the dataset might have missing or erroneously recorded input variables, and some outcome variables were not available as they were not recorded. Additionally, over 6000 newborns were excluded due to missing notes and incomplete data. This exclusion could introduce selection bias, particularly if the excluded newborns differ systematically from those included. Furthermore, outcome extraction relying on ICD coding from EHRs to assign diagnosis dates might not always reflect the exact onset of a condition, which might result in minor information leakage for certain conditions. Future work will also focus on integrating additional data modalities, including continuous cardiorespiratory monitoring, medication records, and other structured data, to improve outcome granularity and enhance the precision of labels for complex neonatal conditions. NeonatalBERT was pre-trained on data from a single health-care system (Stanford Health Care), and clinical documentation and workflows might vary substantially across different health systems. Although NeonatalBERT showed strong performance on an external cohort from the Beth Israel Deaconess Medical Center following model fine-tuning, this does not ensure the same level of performance on clinical notes from different health-care systems. Although NeonatalBERT showed strong performance across two cohorts, the restricted sample size and relatively small proportion of preterm infants constrain generalisability. Future validation in larger, more diverse neonatal populations is needed to verify the model’s generalisability.

Overall, this study shows that a neonatal-specific LLM could leverage routine clinical notes to estimate risk across a broad spectrum of neonatal morbidities in two independent cohorts. Although predictive performance varied by outcome—being descriptive for conditions identifiable at birth and anticipatory for those with later onset—the consistent performance of NeonatalBERT across institutions supports its use as a complementary clinical tool, particularly when structured data are incomplete or unavailable. These findings support the use of automated risk estimation to enable earlier intervention, improve clinical workflows, and enhance communication with families, while highlighting the importance of responsible integration, validation, and governance of AI systems in neonatal care.

## Data sharing

The implementation of NeonatalBERT was done using Python 3.9 and PyTorch, with key libraries including Scikit-learn, Hugging Face Transformers, NumPy, and Pandas for model development and evaluation. All experiments were done on a Linux-based system equipped with 503 GB RAM, dual Intel Xeon Gold 6332 CPUs, and 4 NVIDIA A40 GPUs. The NeonatalBERT model code, including preprocessing scripts and model architecture, is publicly available at https://github.com/fengx13/NeonatalBERT. The primary cohort data from Stanford are not publicly available due to institutional and patient privacy restrictions. We are actively exploring pathways to release a de-identified, pre-trained version of NeonatalBERT that complies with relevant patient privacy regulations and institutional policies. The external cohort data used in this study are publicly available through the Medical Information Mart for Intensive Care III database (https://physionet.org/content/mimiciii/1.4/), subject to credentialled access and a data use agreement.

## Declaration of interests

We declare no competing interests.

## References

[1] Stoll BJ, Hansen NI, Bell EF (2015). Trends in care practices, morbidity, and mortality of extremely preterm neonates, 1993-2012. JAMA.

[2] De Francesco D, Reiss JD, Roger J (2023). Data-driven longitudinal characterization of neonatal health and morbidity. Sci Transl Med.

[3] Mangold C, Zoretic S, Thallapureddy K, Moreira A, Chorath K, Moreira A (2021). Machine learning models for predicting neonatal mortality: a systematic review. Neonatology.

[4] Min B, Ross H, Sulem E (2024). Recent advances in natural language processing via large pre-trained language models: a survey. ACM Comput Surv.

[5] Thirunavukarasu AJ, Ting DSJ, Elangovan K, Gutierrez L, Tan TF, Ting DSW (2023). Large language models in medicine. Nat Med.

[6] Devlin J, Chang M-W, Lee K, Toutanova K (2019). BERT: pre-training of deep bidirectional transformers for language understanding.

[7] Lee J, Yoon W, Kim S (2020). BioBERT: a pre-trained biomedical language representation model for biomedical text mining. Bioinformatics.

[8] Alsentzer E, Murphy J, Boag W (2019). Publicly Available Clinical BERT Embeddings.

[9] Mao C, Xu J, Rasmussen L (2023). AD-BERT: using pre-trained language model to predict the progression from mild cognitive impairment to Alzheimer’s disease. J Biomed Inform.

[10] Collins GS, Moons KGM, Dhiman P (2024). TRIPOD+AI statement: updated guidance for reporting clinical prediction models that use regression or machine learning methods. BMJ.

[11] Johnson AEW, Pollard TJ, Shen L (2016). MIMIC-III, a freely accessible critical care database. Sci Data.

[12] De Francesco D, Blumenfeld YJ, Marić I (2022). A data-driven health index for neonatal morbidities. iScience.

[13] Taha AA, Hanbury A (2015). Metrics for evaluating 3D medical image segmentation: analysis, selection, and tool. BMC Med Imaging.

[14] Politis MD, Bermejo-Sánchez E, Canfield MA (2021). Prevalence and mortality in children with congenital diaphragmatic hernia: a multicountry study. Ann Epidemiol.

[15] Wornow M, Xu Y, Thapa R (2023). The shaky foundations of large language models and foundation models for electronic health records. NPJ Digit Med.

[16] Gekhman Z, Yona G, Aharoni R (2024). Does fine-tuning LLMs on new knowledge encourage hallucinations?.

[17] Dong H, Suárez-Paniagua V, Zhang H (2023). Ontology-driven and weakly supervised rare disease identification from clinical notes. BMC Med Inform Decis Mak.

[18] Chen S, Guevara M, Moningi S (2024). The effect of using a large language model to respond to patient messages. Lancet Digit Health.

[19] Hager P, Jungmann F, Holland R (2024). Evaluation and mitigation of the limitations of large language models in clinical decision-making. Nat Med.

[20] Brown KE, Yan C, Li Z (2025). Large language models are less effective at clinical prediction tasks than locally trained machine learning models. J Am Med Inform Assoc.

[21] Verlingue L, Boyer C, Olgiati L, Brutti Mairesse C, Morel D, Blay J-Y (2024). Artificial intelligence in oncology: ensuring safe and effective integration of language models in clinical practice. Lancet Reg Health Eur.

[22] Jiang LY, Liu XC, Nejatian NP (2023). Health system-scale language models are all-purpose prediction engines. Nature.

[23] Chen KT, Ringer S, Cohen AP, Lieberman E (2002). The role of intrapartum fever in identifying asymptomatic term neonates with early-onset neonatal sepsis. J Perinatol.

[24] Thompson AM, Bizzarro MJ (2008). Necrotizing enterocolitis in newborns: pathogenesis, prevention and management. Drugs.

[25] Razak A, Patel RM, Gautham KS (2021). Use of probiotics to prevent necrotizing enterocolitis: evidence to clinical practice. JAMA Pediatr.

[26] Ballabh P, de Vries LS (2021). White matter injury in infants with intraventricular haemorrhage: mechanisms and therapies. Nat Rev Neurol.

[27] Petersen JD, Lozovatsky M, Markovic D (2020). Clinical decision support for hyperbilirubinemia risk assessment in the electronic health record. Acad Pediatr.

[28] Cavolo A, Dierckx de Casterlé B, Naulaers G, Gastmans C (2022). Neonatologists’ resuscitation decisions at birth for extremely premature infants. A Belgian qualitative study. Front Pediatr.

[29] Vijlbrief D, Dudink J, van Solinge W, Benders M, Haitjema S (2023). From computer to bedside, involving neonatologists in artificial intelligence models for neonatal medicine. Pediatr Res.

[30] Alves M, Seringa J, Silvestre T, Magalhães T (2024). Use of artificial intelligence tools in supporting decision-making in hospital management. BMC Health Serv Res.

[31] Ning Y, Teixayavong S, Shang Y (2024). Generative artificial intelligence and ethical considerations in health care: a scoping review and ethics checklist. Lancet Digit Health.

[32] Lee CR, Aysola J, Chen X (2024). Race and ethnicity and clinician linguistic expressions of doubt in hospital admission notes. JAMA Netw Open.

[33] Carrell DS, Schoen RE, Leffler DA (2017). Challenges in adapting existing clinical natural language processing systems to multiple, diverse health care settings. J Am Med Inform Assoc.

