# Supplementary material for: Development and validation of a pre-trained language model for neonatal morbidities: a retrospective, multicentre, prognostic study
Source: Lancet Digit Health. 2025 Dec 18;7(12):None. doi: 10.1016/j.landig.2025.100926 (PMC12748047; doi:10.1016/j.landig.2025.100926)
Supplement: Supplementary appendix [file mmc1.pdf]

THE LANCET
Digital Health

## Supplementary appendix

This appendix formed part of the original submission and has been peer reviewed.
We post it as supplied by the authors.

Supplement to: Xie F, Chung P, Reiss JD, et al. Development and validation of a pre-trained language model for neonatal morbidities: a retrospective, multicentre, prognostic study. *Lancet Digit Health* 2025. https://doi.org/10.1016/j.landig.2025.100926

# SUPPLEMENTAL MATERIAL

**eTable 1:** List of tabular variables used in logistic regression baselines and definitions of neonatal outcomes for the Stanford data (Primary cohort).

**eTable 2:** List of neonatal outcomes and corresponding International Classification of Diseases (ICD) codes for the Beth Israel Deaconess Medical Center data (External cohort).

**eTable 3:** Comparison of AUROC scores achieved by different models on the testing data from (A) the primary cohort and (B) the external cohort.

**eTable 4:** Comparison of F1 scores achieved by different models on the testing data from (A) the primary cohort and (B) the external cohort.

**eTable 5:** Sensitivity and specificity of NeonatalBERT for predicting neonatal outcomes compared to Meta's conversational large language model (Llama-3.1-8B-Instruct).

**eTable 6:** Prediction performance of NeonatalBERT across Days 1–7 after birth.

**eTable 7:** Prediction performance of NeonatalBERT using reweighting methods to handle imbalanced outcomes.

**eTable 8:** Prediction performance of NeonatalBERT using term frequency-inverse document frequency (TF-IDF) combined with traditional machine learning methods for comparison, including support vector machines (SVMs) and XGBoost.

**eTable 9:** Prediction performance of NeonatalBERT using different truncation and pooling strategies for handling notes exceeding 512 tokens.

**eMethods**: Data processing, model training, implementation and evaluation

**eFigure 1:** t-distributed stochastic neighbor embedding (t-SNE) plots for different neonatal morbidities using NeonatalBERT embeddings for the primary cohort.

**eFigure 2:** t-distributed stochastic neighbor embedding (t-SNE) plots for different neonatal morbidities using NeonatalBERT embeddings for the external cohort.

**eFigure 3:** Comparison of NeonatalBERT's performance using different note types from the primary cohort: (A) Mean AUROC, (B) Mean AUPRC, and (C) Distribution of different note types.

**eFigure 4:** Calibration plots for different neonatal morbidities using NeonatalBERT for the prediction based on primary cohort.

**eTable 1:** List of tabular variables used in logistic regression baselines and definitions of neonatal outcomes for the Stanford data (Primary cohort).

| Tabular Variable Name | Definition |
|---|---|
| Demographics & Administrative | |
| Mother age | Mother age in years, specific to the newborn |
| Gender (newborn) | Newborn gender identity was recorded as a binary variable (male/female) based on documentation in the electronic health record, which reflects the biological sex assigned at birth. |
| Gestational age | length of time, measured in weeks, that a fetus has been developing in the mother's womb since the first day of the mother's last menstrual period. They were obtained through electronic health records or clinician's notes |
| Birth weight | The weight of a newborn measured immediately after birth. They were obtained through electronic health records or clinician's notes |
| Clinical data – Vital measurements | |
| Heart rate, Respiration rate, Diastolic blood pressure, Systolic blood pressure, $SpO_2$, Temperature | Maternal vital measurements extracted from electronic health records data observed at delivery/birth |
| Clinical data – Lab measurements | |
| Alanine Aminotransferase, Albumin, Alkaline phosphatase, Anion gap, Aspartate aminotransferase, Basophils, Bicarbonate, Bilirubin total, Calcium, Carbon dioxide, Chloride, Creatinine, Eosinophils, Erythrocytes, Globulin, Hematocrit, Glucose, Glomerular filtration rate, Hemoglobin A1c, Immature granulocytes, Leukocytes, Neutrophils, Nucleated erythrocytes, Platelets, pH of Urine, Potassium, Urea nitrogen, Monocytes, Nucleated erythrocytes | Maternal lab measurements extracted from electronic health records data observed at delivery/birth |
| Neonatal Outcomes (SNOMED concept codes were extracted from patient medical records with a minimum of 3 months of follow-up and matched to their corresponding neonatal outcomes. A neonatal outcome was deemed to be absent if the representative codes were not identified) | |
| **Respiratory Morbidities** | |
| RDS (respiratory distress syndrome) | 45768986, 45772947, 258866 |
| BPD (bronchopulmonary dysplasia) | 4236182, 42600161, 4263344, 4283942, 4201423, 4263343, 313023 |

| | |
|---|---|
| Pulmonary hemorrhage | 4149586, 42573131, 256036, 195289, 257375, 42536566, 4111119, 4051335, 761075, 4171119, 43021073, 4301606, 42573132, 4071717 |
| Pulmonary hypertension | 44783620, 44783619, 40493243, 44783622, 40482858, 4124831, 4013643, 44782561, 44783625, 44782562, 44783621, 4121462, 44783623, 44783624, 4119611, 4121620, 44783618, 44783626, 44782560, 36715093 |
| Atelectasis | 4278842, 260212, 258554, 4243494, 4006329 |
| MAS (meconium aspiration syndrome) | 4048150, 4173178, 4153454, 4048457, 439934, 437374, 4172995, 433589, 434154 |
| **Neurological Morbidities** | |
| IVH (intraventricular hemorrhage) | 4079973, 42535103, 36716544, 4048279, 4048278, 436519, 4144154, 434155, 4110185, 4171123, 4048277, 4173332, 36716627, 4079972, 4180743, 36716543, 443752, 37394466 |
| PVL (periventricular leukomalacia) | 372435, 4071867 |
| Seizures | 4043411 4046209 762706 4171110 37399364 4159149 762705 37395921 762709 762579 380533 4089691 4186827 36675039 4244383 |
| Other central nervous system disorders | 46273607, 42535008, 42535007, 4061270, 444292, 4318859, 4200079, 36714076, 4290019, 4182388, 377980, 36674814, 42535006, 372444, 4082314, 4318860, 4319463, 4079556, 442631, 42535380 |
| **Cardiac Morbidities** | |
| PDA (patent ductus arteriosus) | 43021583, 37205075, 4053893, 4109328, 37204212, 315922 |
| Cardiovascular instability | 4173170, 37395937, 4106274, 4110550, 443522, 443523 |
| **Infectious Diseases** | |
| Neonatal sepsis | 133594, 4048275, 46270041, 36715567, 761851, 35622880, 42536689, 4071727, 4048594, 35622881, 4071063, 761852, 763027 |
| Candidiasis | 4300236, 440840, 4070650, 42538263, 36717505, 4070651, 4070648 |
| **Blood Disorders** | |
| Anemia | 4339722, 4079852, 4173191, 36713168, 4071073, 36674478, 432452 |
| Jaundice | 4071740, 4067525, 4170445, 4071737, 4071736, 4096143, 4071080, 4251487, |

| | |
|---|---|
| | 440847, 4071741, 4048294, 4239658, 4048290, 4071083, 4048614, 435656, 4071079, 4048293, 4048610, 4230351, 4221399, 4048613, 4328890, 4071735, 4071743, 4165508, 4071076, 4171095, 439137, 4173180 |
| **Other Conditions** | |
| NEC (necrotizing enterocolitis) | 37311911, 4308227, 201957, 37311908, 37311912, 37311909, 37311910, 37311907, 4287783 |
| ROP (retinopathy of prematurity) | 37207968, 36684751, 443520, 36684752, 373766, 36684624, 443519, 375251, 36684621, 36684754, 36684622, 36684753, 36684685, 36684687, 36684684, 36684686, 375250, 36684623, 379009 |
| Death | Death occurring after birth |

**eTable 2:** List of neonatal outcomes and corresponding International Classification of Diseases (ICD) codes for the Beth Israel Deaconess Medical Center data (External cohort).

| Neonatal outcomes | ICD-9-CM code(s) |
|---|---|
| **Respiratory Morbidities** | |
| RDS (respiratory distress syndrome) | 769 |
| BPD (bronchopulmonary dysplasia) | 770.7 |
| Pulmonary hemorrhage | 770.3 |
| Pulmonary hypertension | 416.0 |
| **Neurological Morbidities** | |
| IVH (intraventricular hemorrhage) | 772.1 |
| **Cardiac Morbidities** | |
| PDA (patent ductus arteriosus) | 747.0 |
| **Infectious Diseases** | |
| Neonatal sepsis | 771.81 |
| **Blood Disorders** | |
| Jaundice | 774; 277.4 |
| **Other Conditions** | |
| NEC (necrotizing enterocolitis) | 777.5 |
| ROP (retinopathy of prematurity) | 362.2 |
| Death | Death occurring after birth |

ICD-9-CM: International Classification of Diseases, Ninth Revision, Clinical Modification

**eTable 3:** Comparison of AUROC scores achieved by different models on the testing data from (A) the primary cohort and (B) the external cohort. Bold numbers indicate the best performance in the comparison. Bold numbers indicate the best performance in the comparison.

| **(A) Primary cohort (Stanford data)** | | | | |
|---|---|---|---|---|
| Outcome | NeonatalBERT | Logistic regression using tabular data | Bio-ClinicalBERT | BioBERT |
| **Respiratory Morbidities** | | | | |
| RDS | **0.933** | 0.819 | 0.916 | 0.888 |
| BPD | **0.990** | 0.989 | 0.984 | 0.985 |
| Pulmonary hemorrhage | **0.994** | 0.980 | 0.982 | 0.981 |
| Pulmonary hypertension | **0.921** | 0.520 | 0.910 | 0.910 |
| Atelectasis | **0.905** | 0.721 | 0.853 | 0.859 |
| Aspiration syndrome | **0.865** | 0.660 | 0.814 | 0.761 |
| **Neurological Morbidities** | | | | |
| IVH | **0.964** | 0.880 | 0.958 | 0.961 |
| PVL | **0.958** | 0.735 | 0.809 | 0.912 |
| Seizures | **0.864** | 0.644 | 0.846 | 0.820 |
| Other CNS disorders | **0.950** | 0.568 | 0.934 | 0.930 |
| **Cardiac Morbidities** | | | | |
| PDA | **0.893** | 0.692 | 0.882 | 0.867 |
| Cardiovascular instability | **0.887** | 0.805 | 0.872 | 0.847 |
| **Infectious Diseases** | | | | |
| Neonatal sepsis | **0.863** | 0.778 | 0.862 | 0.830 |
| Candidiasis | **0.700** | 0.577 | 0.659 | 0.612 |
| **Blood Disorders** | | | | |
| Anemia | **0.982** | 0.976 | 0.971 | 0.969 |
| Jaundice | **0.658** | 0.597 | 0.627 | 0.638 |
| **Other Conditions** | | | | |
| NEC | **0.980** | 0.918 | **0.980** | 0.973 |
| ROP | **0.986** | 0.95 | 0.967 | 0.967 |
| Neonatal mortality | **0.989** | 0.916 | 0.974 | 0.955 |
| **Mean Over All Outcomes** | **0.910 (0.900–0.921)** | 0.775 (0.749–0.802) | 0.884 (0.870–0.901) | 0.877 (0.867–0.890) |
| **(B) External Cohort (BIDMC Dataset)** | | | | |
| Outcome | NeonatalBERT | Logistic regression using tabular data | Bio-ClinicalBERT | BioBERT |

| | | | | |
|---|---|---|---|---|
| **Respiratory Morbidities** | | | | |
| RDS | **0.965** | 0.851 | 0.961 | 0.960 |
| BPD | **0.951** | 0.887 | 0.939 | 0.937 |
| Pulmonary hemorrhage | **0.960** | 0.629 | 0.933 | 0.937 |
| **Neurological Morbidities** | | | | |
| IVH | **0.913** | 0.867 | 0.908 | 0.898 |
| **Cardiac Morbidities** | | | | |
| PDA | **0.907** | 0.839 | 0.902 | 0.906 |
| **Infectious Diseases** | | | | |
| Neonatal sepsis | **0.900** | 0.843 | 0.890 | 0.896 |
| **Blood Disorders** | | | | |
| Jaundice | **0.891** | 0.826 | 0.883 | 0.879 |
| **Other Conditions** | | | | |
| NEC | **0.922** | 0.823 | 0.908 | 0.902 |
| ROP | **0.954** | 0.847 | 0.945 | 0.947 |
| Neonatal mortality | **0.986** | 0.752 | 0.977 | 0.986 |
| **Mean Over All Outcomes** | **0.935 (0.928–0.942)** | 0.816 (0.772–0.860) | 0.925 (0.917, 0.932) | 0.925 (0.917, 0.933) |

RDS, respiratory distress syndrome; BPD, bronchopulmonary dysplasia; IVH, intraventricular hemorrhage; PVL, periventricular leukomalacia; PDA, patent ductus arteriosus; CNS, central nervous system; NEC, necrotizing enterocolitis; ROP, Retinopathy of Prematurity

**eTable 4:** Comparison of F1 scores achieved by different models on the testing data from (A) the primary cohort and (B) the external cohort. Bold numbers indicate the best performance in the comparison.

| **(A) Primary cohort (Stanford data)** | | | | |
|---|---|---|---|---|
| Outcome | NeonatalBERT | Logistic regression using tabular data | Bio-ClinicalBERT | BioBERT |
| **Respiratory Morbidities** | | | | |
| RDS | **0.684** | 0.502 | 0.599 | 0.493 |
| BPD | 0.517 | **0.626** | 0.493 | 0.439 |
| Pulmonary hemorrhage | 0.111 | **0.267** | 0.067 | 0.051 |
| Pulmonary hypertension | **0.247** | 0.011 | 0.154 | 0.160 |
| Atelectasis | 0.051 | 0.009 | 0.025 | **0.083** |
| Aspiration syndrome | **0.043** | 0.020 | 0.026 | 0.043 |
| **Neurological Morbidities** | | | | |
| IVH | 0.211 | **0.298** | 0.185 | 0.232 |
| PVL | **0.020** | 0.002 | 0.003 | 0.006 |
| Seizures | 0.183 | 0.008 | **0.318** | 0.222 |
| Other CNS disorders | **0.429** | 0.005 | 0.154 | 0.375 |
| **Cardiac Morbidities** | | | | |
| PDA | **0.518** | 0.051 | 0.430 | 0.516 |
| Cardiovascular instability | **0.461** | 0.390 | 0.364 | 0.294 |
| **Infectious Diseases** | | | | |
| Neonatal sepsis | 0.160 | 0.018 | 0.204 | **0.209** |
| Candidiasis | **0.018** | 0.013 | 0.018 | 0.016 |
| **Blood Disorders** | | | | |
| Anemia | 0.620 | **0.684** | 0.600 | 0.636 |
| Jaundice | **0.623** | 0.602 | 0.608 | 0.615 |
| **Other Conditions** | | | | |
| NEC | 0.095 | 0.144 | **0.167** | 0.125 |
| ROP | 0.576 | **0.594** | 0.430 | 0.418 |
| Neonatal mortality | **0.606** | 0.491 | 0.364 | 0.387 |
| Mean Over All Outcomes | **0.325** | 0.249 | 0.274 | 0.280 |
| **(B) External Cohort (BIDMC Dataset)** | | | | |
| Outcome | NeonatalBERT | Logistic regression using tabular data | Bio-ClinicalBERT | BioBERT |

| | | | | |
|---|---|---|---|---|
| **Respiratory Morbidities** | | | | |
| RDS | **0.825** | 0.602 | 0.805 | 0.805 |
| BPD | **0.422** | 0.195 | 0.348 | 0.314 |
| Pulmonary hemorrhage | **0.130** | 0.004 | 0.046 | 0.061 |
| **Neurological Morbidities** | | | | |
| IVH | **0.386** | 0.253 | 0.361 | 0.278 |
| **Cardiac Morbidities** | | | | |
| PDA | **0.459** | 0.391 | 0.458 | 0.470 |
| **Infectious Diseases** | | | | |
| Neonatal sepsis | **0.311** | 0.305 | 0.300 | 0.198 |
| **Blood Disorders** | | | | |
| Jaundice | **0.782** | 0.704 | 0.777 | 0.779 |
| **Other Conditions** | | | | |
| NEC | **0.073** | 0.049 | 0.066 | 0.143 |
| ROP | **0.463** | 0.151 | 0.376 | 0.365 |
| Neonatal mortality | 0.305 | 0.016 | 0.318 | **0.372** |
| **Mean Over All Outcomes** | **0.416** | 0.267 | 0.386 | 0.379 |

RDS, respiratory distress syndrome; BPD, bronchopulmonary dysplasia; IVH, intraventricular hemorrhage; PVL, periventricular leukomalacia; PDA, patent ductus arteriosus; CNS, central nervous system; NEC, necrotizing enterocolitis; ROP, Retinopathy of Prematurity.

**eTable 5:** Sensitivity and specificity of NeonatalBERT for predicting neonatal outcomes compared to Meta's conversational large language model (Llama-3.1-8B-Instruct). Bold numbers indicate the best performance in the comparison.

| | NeonatalBERT | | | Llama-3.1-8B-Instruct | |
|---|---|---|---|---|---|
| Outcomes | Threshold | Sensitivity | Specificity | Sensitivity | Specificity |
| Candidiasis | 0.50% | **82.35%** | 33.02% | 36.86% | **58.33%** |
| BPD | 7.33% | **97.22%** | **99.24%** | 38.02% | 44.44% |
| ROP | 4.28% | **96.77%** | **97.21%** | 36.36% | 65.08% |
| PVL | 0.05% | **75.00%** | **86.21%** | 37.36% | 75.00% |
| RDS | 9.37% | **66.67%** | **86.89%** | 34.45% | 68.22% |
| IVH | 1.66% | **69.44%** | **95.50%** | 37.28% | 64.86% |
| NEC | 0.69% | **80.00%** | **97.16%** | 35.73% | 60.00% |
| Neonatal mortality | 0.53% | **85.71%** | **88.11%** | 38.66% | 65.63% |
| PDA | 3.28% | **53.24%** | **78.99%** | 33.63% | 66.90% |
| Neonatal sepsis | 2.15% | **65.85%** | **84.97%** | 36.95% | 54.55% |
| Pulmonary hemorrhage | 0.21% | **100.00%** | **95.16%** | 29.07% | 75.00% |
| Pulmonary hypertension | 0.56% | **66.67%** | **79.70%** | 27.31% | 58.06% |
| Jaundice | 50.19% | **40.21%** | **74.59%** | 29.15% | 70.31% |
| Aspiration syndrome | 1.79% | **40.91%** | **82.20%** | 38.17% | 57.45% |
| Atelectasis | 0.88% | **68.18%** | **73.52%** | 31.18% | 69.57% |
| Cardiovascular instability | 7.00% | **67.76%** | **84.48%** | 32.62% | 65.74% |
| Other CNS disorders | 0.08% | **91.67%** | **35.37%** | 31.44% | 75.00% |
| Seizures | 1.04% | **36.84%** | **97.27%** | 30.73% | 66.67% |
| Anemia | 1.71% | **97.75%** | **91.99%** | 36.48% | 73.33% |
| PDA | 3.28% | **53.24%** | **78.99%** | 33.63% | 66.90% |
| Running time[1] | **~ 5 min** | | | ~6 hr | |

[1] **inference for 4910 patients for each outcome in the testing dataset from the primary cohort**

**eTable 6:** Prediction performance of NeonatalBERT across Days 1–7 after birth.

| (A) AUROC | | | | | | | |
|---|---|---|---|---|---|---|---|
| Outcome | **Delivery Day** | Day 2 | Day 3 | Day 4 | Day 5 | Day 6 | Day 7 |
| **Respiratory Morbidities** | | | | | | | |
| RDS | 0.933 | 0.952 | 0.948 | 0.947 | 0.949 | 0.947 | 0.946 |
| BPD | 0.990 | 0.995 | 0.996 | 0.996 | 0.997 | 0.997 | 0.997 |
| Pulmonary hemorrhage | 0.994 | 0.993 | 0.997 | 0.996 | 0.995 | 0.994 | 0.993 |
| Pulmonary hypertension | 0.921 | 0.969 | 0.980 | 0.979 | 0.978 | 0.978 | 0.978 |
| Atelectasis | 0.905 | 0.896 | 0.925 | 0.942 | 0.943 | 0.941 | 0.943 |
| Aspiration syndrome | 0.865 | 0.856 | 0.862 | 0.858 | 0.853 | 0.857 | 0.869 |
| **Neurological Morbidities** | | | | | | | |
| IVH | 0.964 | 0.982 | 0.985 | 0.986 | 0.986 | 0.985 | 0.985 |
| PVL | 0.958 | 0.969 | 0.970 | 0.969 | 0.971 | 0.971 | 0.971 |
| Seizures | 0.864 | 0.922 | 0.922 | 0.954 | 0.956 | 0.960 | 0.951 |
| Other CNS disorders | 0.950 | 0.993 | 0.994 | 0.994 | 0.995 | 0.995 | 0.994 |
| **Cardiac Morbidities** | | | | | | | |
| PDA | 0.893 | 0.922 | 0.934 | 0.937 | 0.939 | 0.942 | 0.945 |
| Cardiovascular instability | 0.887 | 0.904 | 0.917 | 0.927 | 0.929 | 0.930 | 0.931 |
| **Infectious Diseases** | | | | | | | |
| Neonatal sepsis | 0.863 | 0.863 | 0.864 | 0.883 | 0.885 | 0.907 | 0.913 |
| Candidiasis | 0.700 | 0.670 | 0.698 | 0.742 | 0.768 | 0.771 | 0.772 |
| **Blood Disorders** | | | | | | | |
| Anemia | 0.982 | 0.983 | 0.981 | 0.980 | 0.982 | 0.984 | 0.984 |
| Jaundice | 0.658 | 0.706 | 0.726 | 0.753 | 0.772 | 0.780 | 0.787 |
| **Other Conditions** | | | | | | | |
| NEC | 0.980 | 0.986 | 0.986 | 0.987 | 0.988 | 0.989 | 0.990 |
| ROP | 0.986 | 0.993 | 0.994 | 0.995 | 0.995 | 0.995 | 0.996 |
| Neonatal mortality | 0.989 | 0.996 | 0.997 | 0.998 | 0.999 | 0.999 | 0.999 |
| **Mean Over All Outcomes** | **0.910** | **0.924** | **0.930** | **0.938** | **0.941** | **0.943** | **0.944** |

| (B) AUPRC | | | | | | | |
|---|---|---|---|---|---|---|---|
| Outcome | **Delivery Day** | Day 2 | Day 3 | Day 4 | Day 5 | Day 6 | Day 7 |
| **Respiratory Morbidities** | | | | | | | |
| RDS | 0.707 | 0.749 | 0.734 | 0.736 | 0.738 | 0.729 | 0.720 |
| BPD | 0.476 | 0.566 | 0.610 | 0.627 | 0.649 | 0.656 | 0.655 |
| Pulmonary hemorrhage | 0.088 | 0.086 | 0.142 | 0.153 | 0.188 | 0.177 | 0.195 |
| Pulmonary hypertension | 0.151 | 0.252 | 0.244 | 0.252 | 0.253 | 0.264 | 0.268 |
| Atelectasis | 0.060 | 0.073 | 0.081 | 0.082 | 0.091 | 0.098 | 0.125 |
| Aspiration syndrome | 0.097 | 0.126 | 0.141 | 0.103 | 0.098 | 0.099 | 0.101 |
| **Neurological Morbidities** | | | | | | | |
| IVH | 0.180 | 0.193 | 0.343 | 0.366 | 0.370 | 0.450 | 0.460 |
| PVL | 0.014 | 0.014 | 0.014 | 0.014 | 0.014 | 0.014 | 0.015 |
| Seizures | 0.083 | 0.320 | 0.272 | 0.258 | 0.265 | 0.333 | 0.355 |
| Other CNS disorders | 0.208 | 0.434 | 0.455 | 0.461 | 0.452 | 0.486 | 0.477 |
| **Cardiac Morbidities** | | | | | | | |
| PDA | 0.395 | 0.494 | 0.491 | 0.489 | 0.502 | 0.512 | 0.514 |
| Cardiovascular instability | 0.399 | 0.452 | 0.485 | 0.507 | 0.510 | 0.524 | 0.529 |
| **Infectious Diseases** | | | | | | | |
| Neonatal sepsis | 0.159 | 0.183 | 0.205 | 0.225 | 0.240 | 0.237 | 0.256 |
| Candidiasis | 0.014 | 0.014 | 0.017 | 0.022 | 0.026 | 0.023 | 0.021 |
| **Blood Disorders** | | | | | | | |
| Anemia | 0.627 | 0.681 | 0.754 | 0.786 | 0.803 | 0.806 | 0.816 |
| Jaundice | 0.576 | 0.637 | 0.654 | 0.691 | 0.719 | 0.733 | 0.744 |
| **Other Conditions** | | | | | | | |
| NEC | 0.093 | 0.147 | 0.090 | 0.086 | 0.097 | 0.111 | 0.105 |
| ROP | 0.986 | 0.993 | 0.994 | 0.995 | 0.995 | 0.995 | 0.996 |
| Neonatal mortality | 0.648 | 0.764 | 0.832 | 0.858 | 0.892 | 0.899 | 0.891 |
| **Mean Over All Outcomes** | **0.291** | **0.358** | **0.380** | **0.389** | **0.399** | **0.412** | **0.417** |

RDS, respiratory distress syndrome; BPD, bronchopulmonary dysplasia; IVH, intraventricular hemorrhage; PVL, periventricular leukomalacia; PDA, patent ductus arteriosus; CNS, central nervous system; NEC, necrotizing enterocolitis; ROP, Retinopathy of Prematurity.

**eTable 7:** Prediction performance of NeonatalBERT using reweighting methods to handle imbalanced outcomes.

| | AUROC | AUPRC | F1 | AUPRC vs random classifier |
|---|---|---|---|---|
| **Respiratory Diseases** | | | | |
| RDS | 0.932 | 0.703 | 0.687 | 10.984 |
| BPD | 0.990 | 0.434 | 0.5 | 43.400 |
| Pulmonary hemorrhage | 0.994 | 0.088 | 0.111 | 88.218 |
| Pulmonary hypertension | 0.921 | 0.151 | 0.247 | 25.200 |
| Atelectasis | 0.905 | 0.060 | 0.051 | 11.994 |
| Aspiration syndrome | 0.865 | 0.097 | 0.043 | 10.806 |
| **Neurological Diseases** | | | | |
| IVH | 0.962 | 0.164 | 0.219 | 23.429 |
| PVL | 0.964 | 0.015 | 0.027 | 15.000 |
| Seizures | 0.810 | 0.061 | 0.084 | 15.250 |
| Other CNS disorders | 0.944 | 0.190 | 0.414 | 5.429 |
| **Cardiac Diseases** | | | | |
| PDA | 0.893 | 0.377 | 0.512 | 13.464 |
| Cardiovascular instability | 0.889 | 0.403 | 0.468 | 8.060 |
| **Infectious Diseases** | | | | |
| Neonatal sepsis | 0.852 | 0.176 | 0.155 | 19.556 |
| Candidiasis | 0.676 | 0.013 | 0.018 | 1.857 |
| **Blood Disorders** | | | | |
| Anemia | 0.980 | 0.567 | 0.597 | 31.500 |
| Jaundice | 0.658 | 0.574 | 0.623 | 1.335 |
| **Other Conditions** | | | | |
| NEC | 0.978 | 0.081 | 0.085 | 40.500 |
| ROP | 0.985 | 0.510 | 0.539 | 25.500 |
| Neonatal mortality | 0.968 | 0.443 | 0.400 | 88.600 |
| **Mean Over All Outcomes** | 0.903 | 0.269 | 0.304 | 25.267 |

**eTable 8**. Prediction performance of traditional machine learning methods using term frequency-inverse document frequency (TF-IDF), including support vector machines (SVMs) and XGBoost.

| Outcome | NeonatalBERT | | TF-IDF (SVM) | | TF-IDF (XGBoost) | |
|---|---|---|---|---|---|---|
| | AUROC | AUPRC | AUROC | AUPRC | AUROC | AUPRC |
| Respiratory Diseases | | | | | | |
| RDS | 0.933 | 0.707 | 0.656 | 0.141 | 0.666 | 0.202 |
| BPD | 0.990 | 0.476 | 0.516 | 0.009 | 0.880 | 0.062 |
| Pulmonary hemorrhage | 0.994 | 0.088 | 0.666 | 0.001 | 0.858 | 0.006 |
| Pulmonary hypertension | 0.921 | 0.151 | 0.628 | 0.021 | 0.569 | 0.014 |
| Atelectasis | 0.905 | 0.060 | 0.664 | 0.012 | 0.634 | 0.007 |
| Aspiration syndrome | 0.865 | 0.097 | 0.698 | 0.022 | 0.625 | 0.012 |
| Neurological Diseases | | | | | | |
| IVH | 0.964 | 0.180 | 0.651 | 0.019 | 0.633 | 0.024 |
| PVL | 0.958 | 0.014 | 0.711 | 0.001 | 0.566 | 0.001 |
| Seizures | 0.864 | 0.086 | 0.677 | 0.004 | 0.787 | 0.029 |
| Other CNS disorders | 0.950 | 0.208 | 0.729 | 0.012 | 0.713 | 0.013 |
| Cardiac Diseases | | | | | | |
| PDA | 0.893 | 0.395 | 0.500 | 0.044 | 0.735 | 0.093 |
| Cardiovascular instability | 0.887 | 0.399 | 0.551 | 0.090 | 0.596 | 0.053 |
| Infectious Diseases | | | | | | |
| Neonatal sepsis | 0.863 | 0.159 | 0.557 | 0.013 | 0.691 | 0.022 |
| Candidiasis | 0.700 | 0.014 | 0.538 | 0.008 | 0.532 | 0.008 |
| Blood Disorders | | | | | | |
| Anemia | 0.982 | 0.627 | 0.629 | 0.032 | 0.635 | 0.044 |
| Jaundice | 0.658 | 0.576 | 0.579 | 0.471 | 0.481 | 0.393 |
| Other Conditions | | | | | | |
| NEC | 0.980 | 0.093 | 0.856 | 0.009 | 0.730 | 0.013 |
| ROP | 0.986 | 0.555 | 0.577 | 0.050 | 0.606 | 0.044 |
| Neonatal mortality | 0.989 | 0.648 | 0.710 | 0.297 | 0.570 | 0.012 |
| Mean Over All Outcomes | **0.910** | **0.291** | **0.636** | **0.066** | **0.658** | **0.055** |

**eTable 9:** Prediction performance of NeonatalBERT using different truncation and pooling strategies for handling notes exceeding 512 tokens.

| Outcome | NeonatalBERT | | | | | |
|---|---|---|---|---|---|---|
| | Truncation without pooling | | Max Pooling | | Mean Pooling | |
| | AUROC | AUPRC | AUROC | AUPRC | AUROC | AUPRC |
| **Respiratory Diseases** | | | | | | |
| RDS | 0.933 | 0.707 | 0.943 | 0.701 | 0.945 | 0.759 |
| BPD | 0.990 | 0.476 | 0.992 | 0.547 | 0.993 | 0.476 |
| Pulmonary hemorrhage | 0.994 | 0.088 | 0.966 | 0.025 | 0.992 | 0.064 |
| Pulmonary hypertension | 0.921 | 0.151 | 0.921 | 0.114 | 0.932 | 0.150 |
| Atelectasis | 0.905 | 0.060 | 0.906 | 0.060 | 0.918 | 0.067 |
| Aspiration syndrome | 0.865 | 0.097 | 0.842 | 0.141 | 0.869 | 0.110 |
| **Neurological Diseases** | | | | | | |
| IVH | 0.964 | 0.180 | 0.970 | 0.189 | 0.977 | 0.209 |
| PVL | 0.958 | 0.014 | 0.928 | 0.007 | 0.962 | 0.013 |
| Seizures | 0.864 | 0.086 | 0.865 | 0.139 | 0.853 | 0.094 |
| Other CNS disorders | 0.950 | 0.208 | 0.947 | 0.249 | 0.976 | 0.413 |
| **Cardiac Diseases** | | | | | | |
| PDA | 0.893 | 0.395 | 0.879 | 0.371 | 0.890 | 0.425 |
| Cardiovascular instability | 0.887 | 0.399 | 0.885 | 0.393 | 0.893 | 0.451 |
| **Infectious Diseases** | | | | | | |
| Neonatal sepsis | 0.863 | 0.159 | 0.858 | 0.085 | 0.872 | 0.150 |
| Candidiasis | 0.700 | 0.014 | 0.721 | 0.016 | 0.692 | 0.014 |
| **Blood Disorders** | | | | | | |
| Anemia | 0.982 | 0.627 | 0.977 | 0.602 | 0.984 | 0.623 |
| Jaundice | 0.658 | 0.576 | 0.647 | 0.562 | 0.652 | 0.573 |
| **Other Conditions** | | | | | | |
| NEC | 0.980 | 0.093 | 0.979 | 0.084 | 0.985 | 0.156 |
| ROP | 0.986 | 0.555 | 0.983 | 0.460 | 0.991 | 0.621 |
| Neonatal mortality | 0.989 | 0.648 | 0.991 | 0.629 | 0.996 | 0.691 |
| **Mean Over All Outcomes** | **0.910** | **0.291** | **0.905** | **0.283** | **0.914** | **0.319** |

**eMethods: Data processing, model training, implementation and evaluation**

**Model development and validation**
The data were analyzed using Python, version 3.9.16, and R software, version 4.2.1 (R Foundation for Statistical Computing).

Our pre-training algorithm builds upon BERT(1), a contextual word representation model using a multilayer bidirectional transformer encoder architecture, originally trained on general-domain texts. Prior work continued pre-training BERT on large-scale biomedical corpora sourced from PubMed abstracts and full texts to yield BioBERT(2), which was further adapted to the clinical domain by pre-training on 2 million clinical notes to yield Bio-ClinicalBERT(3). Starting from Bio-ClinicalBERT, we perform domain-adaptive pretraining(4) with a corpus of neonatal clinical notes obtained during the first 3 months of birth from the primary cohort training set to contextualize the model to the language of neonatal medicine. The domain-adaptive pretraining uses the same self-supervised objectives as the original BERT model, which includes masked language modeling and next-sentence prediction.

We also explored fine-tuning NeonatalBERT to include clinical notes up to Day 7 post-birth to explore longitudinal trends of the predictive performance over time. Pre-training and fine-tuning were implemented using the Huggingface Transformers(5) package, version 4.25.1 and based on PyTorch(6), version 2.0.1. The NeonatalBERT pretraining took 10 days of computational runtime using one NVIDIA A40.

The NeonatalBERT embeddings were obtained for each newborn by getting the final hidden state of the NeonatalBERT model before the logistic classification layer. We also performed t-distributed stochastic neighbor embedding (tSNE) analysis(7), a machine learning algorithm for nonlinear dimensionality reduction, to visualize the NeonatalBERT embeddings based on notes from both cohorts and illustrate how different samples were separated.

The predictive performance of the NeonatalBERT model was reported based on the testing set from both primary and external cohorts. Baseline comparison models were created for each cohort using logistic regression on only tabular EHR features from the first day of birth, including demographics (e.g., birth weight, gestational age, and sex), vital signs (e.g., oxygen saturation, heart rate, respiratory rate), and key laboratory test results (e.g., CRP levels, white blood cell count). The list of tabular variables is given in the Supplemental eTable 1. We also compared the NeonatalBERT with the previously developed BERT-based language models, including BioBERT and Bio-ClinicalBERT. The baseline models were trained and evaluated on the same patients and their notes in each cohort as those used to fine-tune NeonatalBERT. We also included the comparison with state-of-the art conversation-based LLMs to demonstrate the better usability of our model in clinical risk estimation and decision-making support, where Meta's Llama-3.1-8B(8) were included for comparison. The notes and prompt were input, and the sensitivity and specificity were calculated for comparison based on the output of the Llama-3.1-8B. Structured variables with missing values were imputed using training-set medians. Text notes were included as-is, and their absence reflects typical documentation gaps.

**Prediction framework and timepoint**

To ensure alignment with real-world clinical practice, we carefully defined a prediction window to mitigate potential data leakage. The primary prediction point is on the same day when the babies were born. Clinical notes included in the model for training and evaluation were restricted to the delivery day prior to the neonatal diseases were officially diagnosed. Cases where diagnoses occurred on the first day of life were excluded from analysis to avoid overlap between input features and the target variable. Despite these measures, we acknowledge that the date of ICD coding may not perfectly correspond to the true onset of conditions. ICD diagnosis codes were used solely to define outcomes and were not included in the input features for model training or prediction.

The model utilized early clinical notes, including those documented within the first hour or day of life, to predict outcomes with high clinical relevance. These notes often contain baseline neonatal information, such as gestational age, birth weight, APGAR scores, and maternal health history, which are critical for estimating the risk of future conditions.

**Implementation details of NeonatalBERT Pretraining and fine-tuning**

NeonatalBERT was developed by adapting the Bio-ClinicalBERT transformer architecture through additional pretraining on large-scale neonatal-specific clinical notes. This pretraining step allowed the model to learn the specialized language and context unique to neonatal care, enhancing its ability to process complex and unstructured data from newborn documentation. Fine-tuning was performed using notes from the above defined prediction window, with 19 neonatal outcomes as target labels. To address the significant class imbalance inherent in neonatal outcomes, a weighted loss function was implemented, emphasizing underrepresented conditions such as NEC, PVL, and candidiasis. The class weights were manually fine-tuned for each morbidity label within the training set, thereby assigning greater penalty to misclassifications of underrepresented conditions. The model's output embeddings were passed through a classification layer to generate probability distributions for each outcome.

The pre-trained NeonatalBERT was applied to each note to generate note embeddings. For patients with multiple notes within the prediction timeline, we then used a pooling approach to aggregate the note embeddings of a patient by calculating the mean value of each dimension to create a single patient-level representation. Clinical notes exceeding 512 tokens were default truncated to include only the initial 512 tokens, which typically contain the most critical and relevant information in clinical documentation. Additionally, we also provided alternative pooling strategies, such as MeanPooling and MaxPooling to aggregate information from notes exceeding 512 tokens. We first cut long notes into 512-token sections and then calculate the max or mean value for of each dimension. These approaches ensure that the model can effectively handle multiple sources of textual data while adhering to BERT's input constraints.

To optimize model performance, hyperparameters such as learning rate, batch size, and weight decay were tuned through manual grid search using a subset from training data for validation. Early stopping criteria were applied to prevent overfitting during training.

**Model evaluation and calibration**
Model performance was measured using the area under the receiver operating characteristic curve (AUROC) and the area under the precision-recall curve (AUPRC). While AUROC is based on recall and true positive rate, AUPRC is based on recall and positive predictive value, which are not affected by unbalanced outcomes. Hence, AUPRC is more representative when outcomes are rare and unbalanced, like most neonatal outcomes. For visualization purposes, we reported the relative increase/decrease of the AUPRC obtained by the model compared with the AUPRC of a no-skill random classifier, as the reference value for AUPRC depends on the prevalence of the outcome. Considering the imbalance of most neonatal outcomes, the F1 score was calculated to provide another meaningful assessment. Bootstrapped samples were applied to calculate 95% confidence interval (CI).

To assess the calibration of NeonatalBERT across different outcomes, we generated calibration plots by comparing predicted probabilities with observed outcome frequencies. For each outcome, we extracted the predicted probabilities and the corresponding ground-truth binary outcomes from the primary cohort. Predictions were grouped into ten bins based on deciles of the predicted probabilities. Within each bin, we calculated the average predicted probability and the observed event rate. These values were then used to construct calibration curves, with the mean predicted probability on the x-axis and the observed proportion on the y-axis. A dashed 45-degree reference line was included to indicate perfect calibration. Bins with no observations were automatically excluded. This approach allowed for consistent evaluation of calibration performance across multiple outcomes.

To compare performance across different model architectures, we evaluated both NeonatalBERT and Llama-3 on neonatal outcome prediction tasks. NeonatalBERT was fine-tuned on task-specific training data, allowing it to adapt to domain-specific terminology and outcome labels. In contrast, Llama-3 was evaluated in a zero-shot setting without fine-tuning, which aligns with how large generative models are typically accessed and used in practice due to their substantial computational requirements. This comparison reflects a realistic scenario in which domain-specific, fine-tuned models like NeonatalBERT offer practical advantages in accuracy and interpretability over general-purpose models that are often limited to zero-shot inference in clinical settings.

**Implementation of Llama-3.1-8B**
To evaluate the NeonatalBERT against a state-of-the-art conversational large language model (LLM), we implemented Llama-3.1-8B-Instruct as a comparative baseline. Llama-3.1-8B-Instruct is an open-source transformer model with 8 billion parameters developed by Meta. Clinical notes were uniformly preprocessed, and tokenization was performed using Llama-3's native tokenizer to ensure consistency.

For this comparison, Llama-3.1-8B-Instruct was used in inference mode to predict various neonatal outcomes. To constrain the model's responses and ensure structured output, the **Outline** package was employed. This approach provided a controlled format for the predictions generated by the model.

Computational cost was an important consideration in the comparison. Llama-3.1-8B-Instruct required substantial memory for inference and exhibited significantly slower processing speeds (~6 hours to generate predictions for all patients in the primary testing dataset for a single outcome in our computational settings) compared to NeonatalBERT (~5 minutes for the same task). This computational overhead highlights the limitations of Llama-type LLMs in real-world clinical settings, particularly in resource-constrained environments where rapid, efficient inference is critical for timely decision-making.

Below is an example of the prompts used for neonatal outcome prediction with Llama-3.1-8B-Instruct:

*Input:*
Neonatal H&P Notes
Gestational Age: 25w2d
Maternal age: 38-year-old
Maternal Labs: …..
Information for patients: ….
Pregency Summary: ….
Delivery Information: …
Physical Exam: …
….

Will this neonatal patient develop sepsis? Answer 1 if yes, 0 if no. Answer with only the number.

*Output for Llama3.1-8B:*
1

**eFigure 1:** t-distributed stochastic neighbor embedding (t-SNE) plots for different neonatal morbidities using NeonatalBERT embeddings for the primary cohort. Each dot represents one newborn. Blue-color dots: patient without disease. Red-color dots: patient with disease

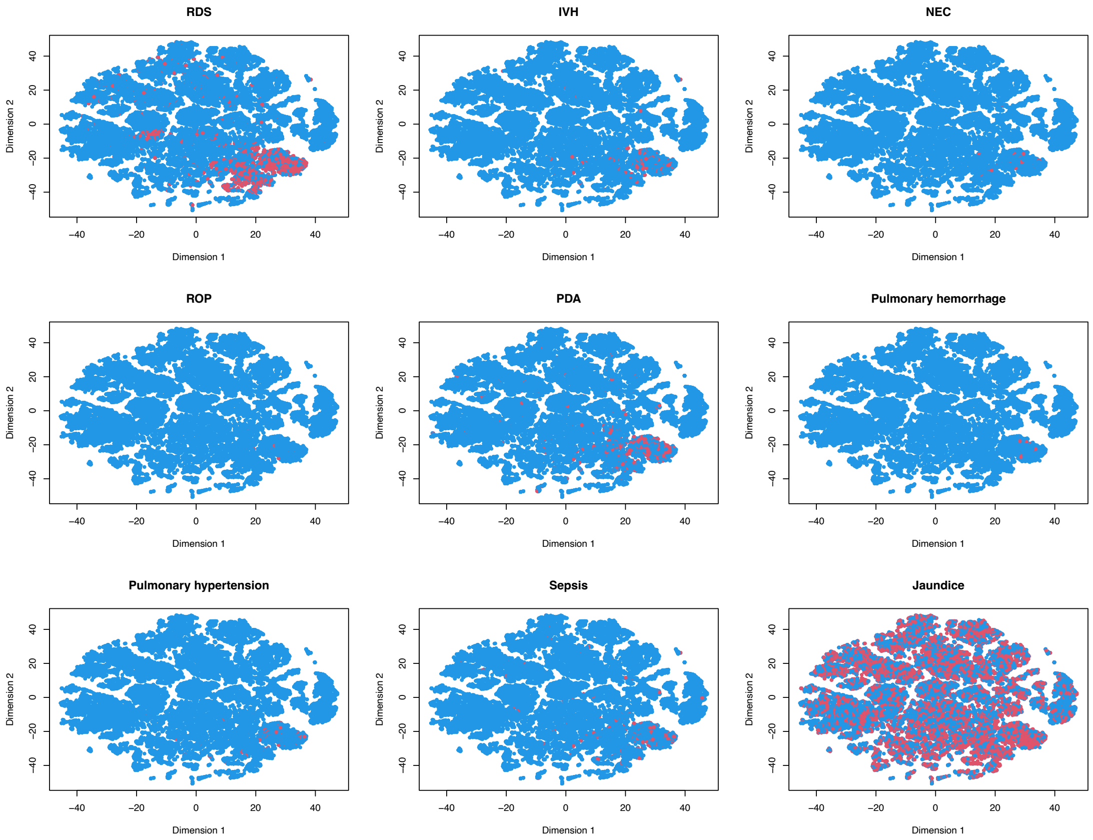


RDS, respiratory distress syndrome; IVH, intraventricular hemorrhage; PDA, patent ductus arteriosus; NEC, necrotizing enterocolitis; ROP, Retinopathy of Prematurity.

**eFigure 2:** t-distributed stochastic neighbor embedding (t-SNE) plots for different neonatal morbidities using NeonatalBERT embeddings for the external cohort. Each dot represents one newborn. Blue-color dots: patient without disease. Red-color dots: patient with disease

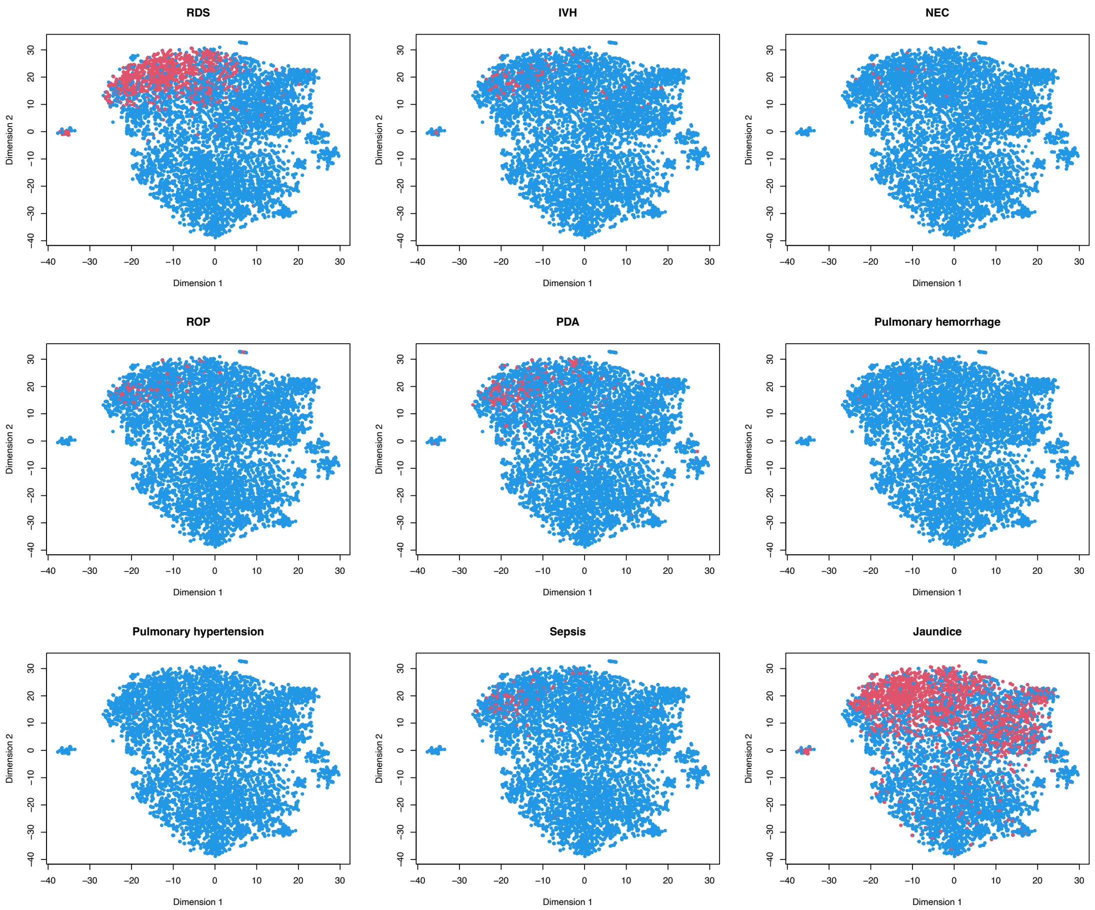


RDS, respiratory distress syndrome; IVH, intraventricular hemorrhage; PDA, patent ductus arteriosus; NEC, necrotizing enterocolitis; ROP, Retinopathy of Prematurity

**eFigure 3:** Comparison of NeonatalBERT's performance using different note types from the primary cohort: (A) Mean AUROC, (B) Mean AUPRC, and (C) Distribution of different note types.

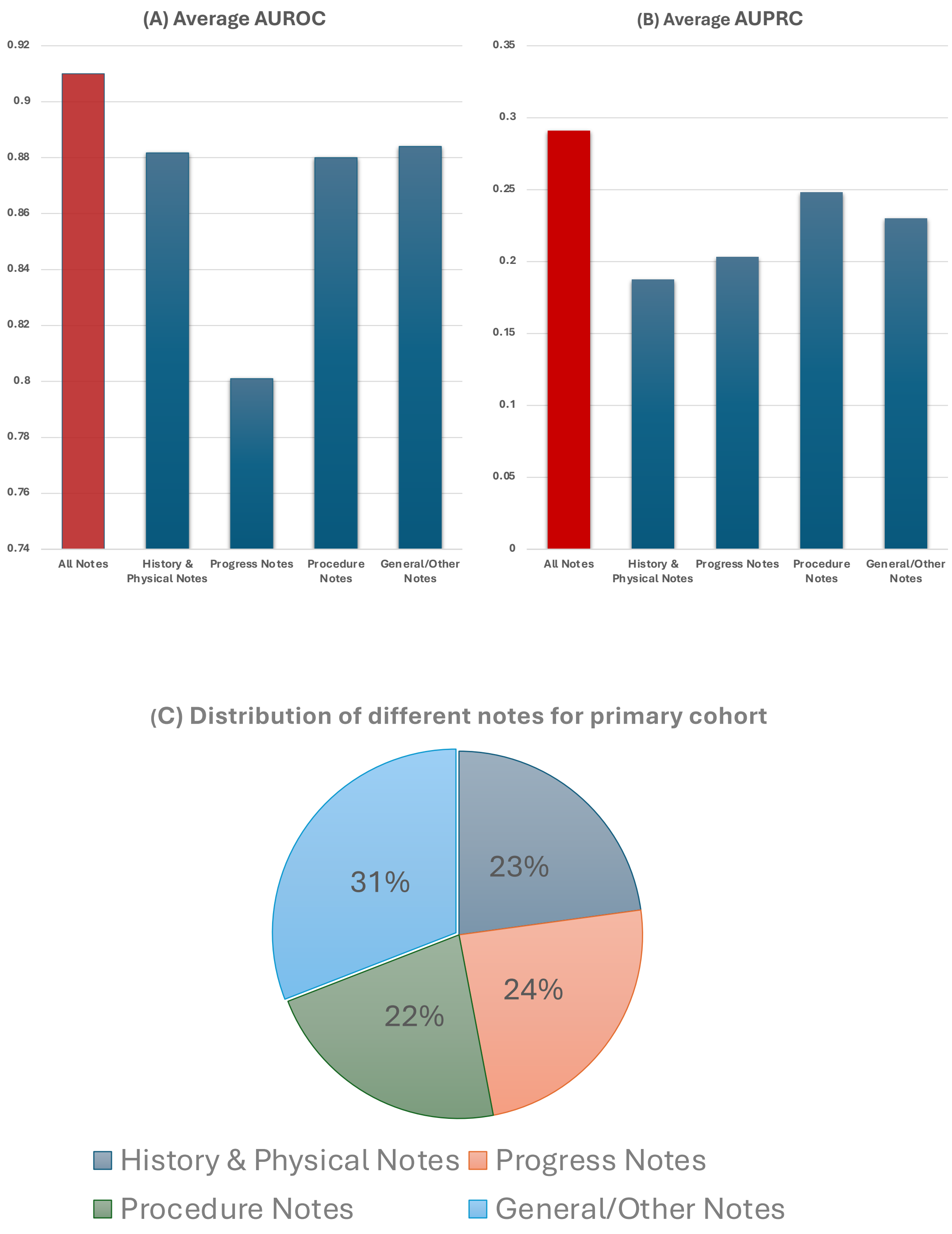

**eFigure 4:** Calibration plots for different neonatal morbidities using NeonatalBERT for the prediction based on primary cohort.

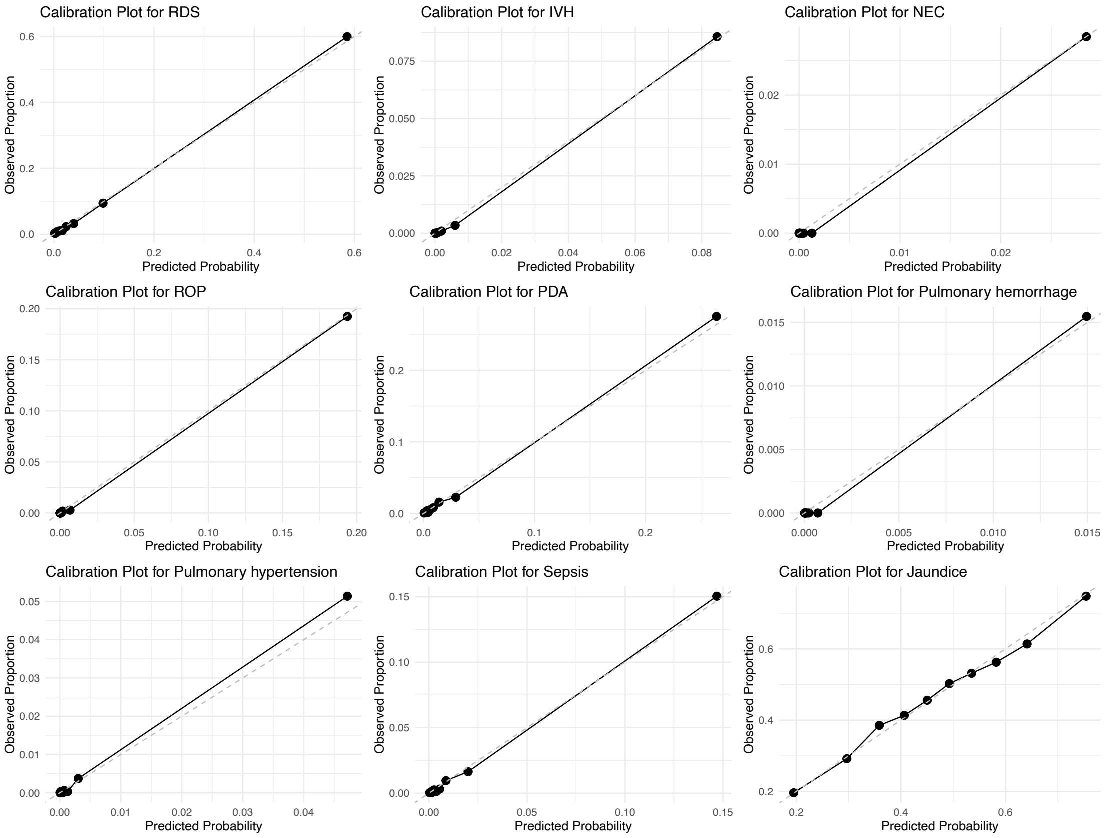


RDS, respiratory distress syndrome; IVH, intraventricular hemorrhage; PDA, patent ductus arteriosus; NEC, necrotizing enterocolitis; ROP, Retinopathy of Prematurity

## Reference:


1. Devlin J, Chang M-W, Lee K, Toutanova K. Bert: Pre-training of deep bidirectional transformers for language und erstanding. arXiv preprint arXiv:181004805.
2. Lee J, Yoon W, Kim S, Kim D, Kim S, So CH, et al. BioBERT: a pre-trained biomedical language representation model for bi omedical text mining. Bioinformatics.36(4):1234-40.
3. Alsentzer E, Murphy J, Boag W, Weng W-H, Jindi D, Naumann T, et al., editors. Publicly Available Clinical BERT Embeddings2019; Minneapolis, Minnesota, USA: Association for Computational Linguistics.
4. Mao C, Xu J, Rasmussen L, Li Y, Adekkanattu P, Pacheco J, et al. AD-BERT: Using pre-trained language model to predict the progression f rom mild cognitive impairment to Alzheimer's disease. Journal of Biomedical Informatics.144:104442.
5. Wolf T, Debut L, Sanh V, Chaumond J, Delangue C, Moi A, et al. Huggingface's transformers: State-of-the-art natural language processing. arXiv preprint arXiv:191003771. 2019.
6. Paszke A, Gross S, Massa F, Lerer A, Bradbury J, Chanan G, et al. Pytorch: An imperative style, high-performance deep learning library. Advances in neural information processing systems. 2019;32.
7. Hinton G, van der Maaten L. Visualizing data using t-SNE. J Mach Learn Res. 2008;9(Nov):2579-605.
8. Dubey A, Jauhri A, Pandey A, Kadian A, Al-Dahle A, Letman A, et al. The llama 3 herd of models. arXiv preprint arXiv:240721783. 2024.
